# Host centric drug repurposing for viral diseases

**DOI:** 10.1371/journal.pcbi.1012876

**Published:** 2025-04-02

**Authors:** Suzana de Siqueira Santos, Haixuan Yang, Aldo Galeano, Alberto Paccanaro

**Affiliations:** 1 Escola de Matemática Aplicada, Fundação Getúlio Vargas, Rio de Janeiro, Brazil; 2 School of Mathematical & Statistical Sciences, University of Galway, Galway, Ireland; 3 Department of Computer Science, Centre for Systems and Synthetic Biology, Royal Holloway, University of London, Egham Hill, Egham, United Kingdom; Pennsylvania State University, UNITED STATES OF AMERICA

## Abstract

Computational approaches for drug repurposing for viral diseases have mainly focused on a small number of antivirals that directly target pathogens (virus centric therapies). In this work, we combine ideas from collaborative filtering and network medicine for making predictions on a much larger set of drugs that could be repurposed for host centric therapies, that are aimed at interfering with host cell factors required by a pathogen. Our idea is to create matrices quantifying the perturbation that drugs and viruses induce on human protein interaction networks. Then, we decompose these matrices to learn embeddings of drugs, viruses, and proteins in a low dimensional space. Predictions of host-centric antivirals are obtained by taking the dot product between the corresponding drug and virus representations. Our approach is general and can be applied systematically to any compound with known targets and any virus whose host proteins are known. We show that our predictions have high accuracy and that the embeddings contain meaningful biological information that may provide insights into the underlying biology of viral infections. Our approach can integrate different types of information, does not rely on known drug-virus associations and can be applied to new viral diseases and drugs.

## Author summary

Drug repurposing is the re-use of de-risked compounds in humans for new therapeutic indications. Computational approaches can help in this process by providing a ranking of compounds with potential effect against a given disease. For viral diseases, computational methods have mainly focused on a small number of antivirals that directly target pathogens (virus centric therapies). Another type of antiviral drugs is aimed at disrupting host cellular processes required for the viral infection, either directly or indirectly (host centric therapies). In this work, we propose a novel computational approach focused on host centric therapies that can make predictions on a large set of drugs. It relies on the human protein-protein interaction network for identifying drugs that may influence host cellular processes required for the viral infection, together with machine learning techniques to enhance prediction accuracy by integrating information across different viruses and drugs. We obtained predictions of drug efficacy for 143 viruses. We show that our method successfully identifies drugs with known evidence against viruses among the top-ranked predictions. Furthermore, our model captures meaningful biological information, providing insights into the underlying biology of viral infections.

## Introduction

Viral infections cause public health crises and numerous deaths across the world. COVID-19 alone is thought to have been responsible for more than 6.9 million deaths worldwide between December 2019 and September 2023 [1]. Developing new drugs against viruses is a challenging, expensive and time-consuming task. Drug repurposing, the re-use of de-risked compounds in humans for new therapeutic indications, may result in shorter development times and lower costs.

Machine learning methods, and in particular collaborative filtering methods based on matrix decomposition, have been developed for the problem of predicting drugs that can be repurposed for non-infectious diseases [2–4]. These methods predict drugs based on similarities in their pattern of effectiveness on a set of diseases. A few attempts have been made to develop this type of methods, as well as other supervised learning approaches, for drug repurposing for viral diseases. These techniques can recommend, for a given viral disease, drugs that are already known to have efficacy against other viral diseases, which, with very few exceptions, are drugs that directly target the viruses [5,6] (virus centric therapies). These approaches are robust, as they combine knowledge about the effectiveness of drugs on different viruses. However, the number of virus centric drugs is still relatively small and therefore the possibilities for repurposing are very limited – there are only 45 drugs of this type approved as antivirals in DrugVirus.Info, one of the largest resources for the exploration and analysis of approved or experimental broad-spectrum antiviral drugs [7].

Another type of antiviral drugs is aimed at interfering with host cellular processes required for the viral infection, either directly or indirectly (host centric therapies) [8–11]. Drug repurposing for host centric therapies could be applied to a much larger set of drugs, potentially to any approved drug or compound available on the market. Drug repurposing for host centric therapies has also been strongly advocated as these therapies are less prone to resistance than those that target pathogens directly [8,9,12–14]. However, machine learning approaches do not seem viable for this problem because of the even smaller number of approved host centric drugs that can be used for training – DrugVirus.Info [7] contains only 5 host centric drugs approved as antivirals.

Recently, a few methods have appeared that exploit ideas from network medicine for drug repurposing in the context of viruses [5,15,16]. Network medicine based approaches were originally developed for genetic diseases [17,18] and they rely on the fact that proteins involved in a disease form a module in the human interactome [19]. Working on COVID-19, Gysi et al. [15], Fiscon et al. [16], and Santos et al. [5] realized that the set of human proteins that the virus used for replicating itself after entering the human cell (*host proteins*) also constitutes a module in the interactome. When a drug binds to its targets it causes a perturbation that is propagated through the interactome. Gysi et al. [15] and Santos et al. [5] were able to calculate the extent of the perturbation that a drug induces on the host protein module and thus its predicted effectiveness against the virus – that is, they could predict host centric antivirals for COVID-19. However, while these methods were applicable to any approved drug with known targets, their predictions were noisy due to our incomplete knowledge of the human interactome, drug targets, and sets of host proteins.

These two types of computational approaches seem to be complementary: collaborative filtering methods based on matrix decomposition can combine knowledge about the effectiveness of drugs for different viruses, and this makes them robust, but they are only applicable to a few drugs; network medicine-based approaches can make predictions for any drug with known targets, but are very noisy, and they could benefit from integrating knowledge about other drugs and viruses. In this work, we propose an approach that combines ideas from both areas for predicting host-centric antivirals. We use ideas from network medicine to infer matrices representing how perturbations from drug targets and host proteins propagate on the human protein-protein interaction network (PPI) – this can be done for any approved drug or compound with known targets. We then decompose these matrices to embed drugs, viruses, and proteins into a common low dimensional space – this effectively integrates knowledge about different drugs and viruses. Predictions for drug repurposing are then obtained by simply taking dot products between these embeddings, thus facilitating interpretability. We show that our predictions are accurate and that the learned representations for drugs, viruses, and proteins encode meaningful biological information, such as membership to metabolic pathways, protein function, drug chemical structure, and drug Anatomical Therapeutic Chemical (ATC) category. Our approach is general, it can be applied to new viral diseases and drugs, and other types of information can be included in the inference process – we showcase this by integrating transcriptomics information about viral diseases.

## Results

Host centric therapies aim at interfering with host cellular processes required for viral infection. In other words, given a virus, these therapies attempt to perturb the area in the human interactome that is also perturbed by the viral infection. Our key idea to combine network medicine and collaborative filtering is to learn embeddings for drugs and viruses in a latent space such that their representation is similar when they perturb similar areas in the interactome. In this way, prediction scores for host-centric antivirals can be obtained by simply taking the dot product between the corresponding drug and virus representations. Our assumption is that drugs, viruses and proteins in the interactome can all be represented as latent feature vectors (or signatures) in a low-dimensional space of size *k*, and that these latent features vectors capture molecular or cellular mechanisms related to the effect of drugs and viral infections.

**Fig 1:**
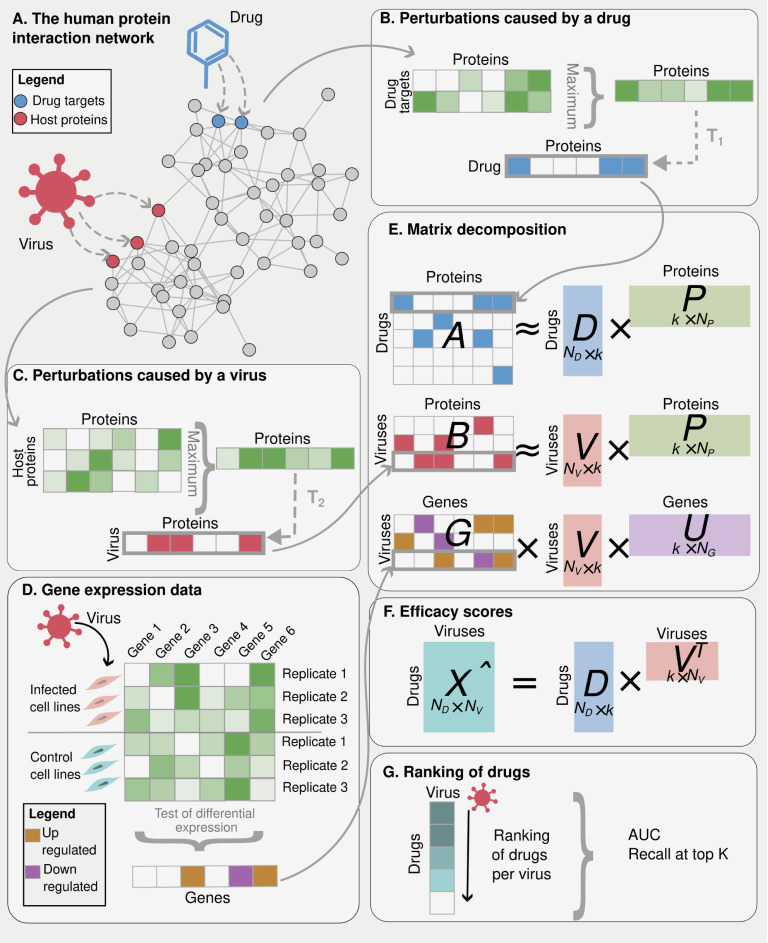
Overview of our approach. **A) The human protein interaction network** containing host proteins (red) and drug targets (blue) for a given virus and drug, respectively. **B) Perturbation caused by a drug.** Given a drug, to quantify the perturbation that it causes on a protein in the PPI network, we calculated the maximum value of the 2-step random walk kernel between each drug target and the protein. For each drug, we then binarized the values obtained for every protein in the PPI with threshold $T_{1}$, and stored as a row in *A*. **C) Perturbation caused by a virus.** Given a virus, to quantify the perturbation that it causes on a protein in the PPI network, we calculated the maximum value of the 2-step random walk kernel between each viral host protein and the protein. For each virus, we then binarized the values obtained for every protein in the PPI with threshold $T_{2}$, and stored as a row in *B*. **D) Gene expression data.** Given a virus, we tested whether genes were differentially expressed between infected cell lines (or tissues) and control cell lines (or tissues). We then created a vector of 0s, 1s and -1s corresponding to a row in matrix *G*, where 0 indicates no significant difference in expression, 1 indicates up-regulation in the infection, and -1 indicates down-regulation. **E) Matrix decomposition.** We decompose the three matrices *A*, *B* and *G* simultaneously, by minimizing Eq. 1. These decompositions provide us with an embedding for each drug, virus and gene in a low dimensional space of size *k*. Note that matrices P and V are shared, thus constraining the three decompositions. **F) Efficacy scores**. We obtain drug efficacy scores by taking the dot product between drug (*D*) and virus (*V*) representations in the low dimensional space. **G) Ranking of drugs**. For each virus, we obtain a ranking of drugs according to their predicted efficacy. In our training set, *n*_*D*_ = 2,197, *n*_*V*_ = 143, *n*_*P*_ = 17,644, *n*_*G*_ = 17,977.

Fig 1 presents an outline of our method. The signatures are obtained by decomposing matrices that encode the network medicine notion of perturbations caused by drugs and viruses on the interactome. These are matrix $A\left(n_{D}\times n_{P}\right)$ and matrix $B\left(n_{V}\times n_{P}\right)$ in Fig 1e, and they are obtained by diffusing drug targets and host proteins on the PPI, respectively (Fig 1a) – they quantify how much drugs and viruses perturb the human protein interaction network. The rows of *A* and *B* correspond to $n_{D}$ drugs, and $n_{V}$ viruses, respectively, while their columns refer to the same set of $n_{P}$ proteins on the PPI. By decomposing A, we obtain a representation for each drug and each protein in a *k* -dimensional space (rows of $n_{D}\times k$ matrix *D* and columns of $k\times n_{P}$ matrix *P* respectively, Fig 1e). By decomposing *B* we obtain a representation for each virus and each protein in the same *k* -dimensional space (rows of $n_{V}\times k$ matrix *V* and columns of *P* respectively, Fig 1e). Importantly, matrices *A* and *B* are decomposed simultaneously and the fact that they share the same set of proteins constrains the decompositions – note that the matrix *P* in Fig 1e is shared between the two decompositions. In other words, we impose the constraint that the protein representations obtained by decomposing *A* is the same as the representations obtained when decomposing *B*, and these shared representations ensure the integration of the data that appear in *A* and *B*. Also note that all the values in *A* and *B* are non-negative, and we choose to impose matrices *D* and *P* to be also non-negative as this has been shown to lead to more interpretable latent features [20].

The quality of the signatures can be improved by adding further biological knowledge about the processes involved in the infection. This knowledge can be included in our framework as further constraints to our decomposition, and we showcase this for gene expression data. Gene expression profiles of viral infections are often available, and they are informative of the effects of the infection on human cells. Moreover, we can assume that viruses that cause a similar effect on gene expression in human cells are more likely to respond similarly to a treatment. To integrate gene expression information, we learn representations of genes as vectors in *the same* low-dimensional space as drugs, viruses, and proteins. As before, data integration takes place through matrix sharing between different decompositions. This is done by decomposing a third ${\text{n}}_{\text{V}}\times {\text{n}}_{G}$ matrix *G* (Fig 1e), which encodes how much viruses perturb the transcriptome (Fig 1d). The rows of *G* refer to the same ${\text{n}}_{V}$ viruses on matrix *B*, and the columns correspond to ${\text{n}}_{G}$ genes on the gene expression data. This matrix is obtained by analysing differences in gene expression during the infection: the $G_{ij}$ entry of *G* is assigned a value of 0, 1, or −1. A zero indicates no significant difference in expression or missing data, and the values 1 and −1 indicate gene up-regulation, or gene down-regulation, respectively. By decomposing *G*, we obtain a representation for each virus and each gene in the same *k* -dimensional space (rows of *V* and columns of $k\times {\text{n}}_{\text{G}}$ matrix *U* respectively, Fig 1e). Notice that the virus representation (*V*) is shared between the decompositions of *B* and *G*. In this way, we constrain the virus representations so that they tend to be similar for viral diseases with similar gene expression profiles. Also, values in *V* are non-negative to facilitate feature interpretability [20].

Our final predictions (matrix **X̂** available in S1 File) are obtained by taking the dot product between the drug and virus representations in the latent space (Fig 1f). Notice this is fundamentally different from what is done in usual matrix decomposition-based collaborative filtering for recommendation systems [2–5]. In fact, these models obtain predictions for a given association matrix (e.g., drug-virus associations) by decomposing it into lower dimensionality matrices and then recombining them by taking their dot product – that is, the matrices being multiplied are the result of decomposing the original association matrix. Here, we obtain predictions by multiplying matrices that are learned from different decompositions. For this reason, our predictions will be correct only if the representations of viruses and drugs are related to the underlying mechanisms of action of drugs and viruses. In other words, our latent features need to be biologically meaningful and compatible – for example, representing biological processes involved in viral diseases and drug effects. Indeed, in the biological interpretation section, we show that this is the case.

Computationally, all latent features (D,V,P,U) are learned jointly by minimizing the following cost function:


$$f\left(D,P,V,U\right)={\left\|A-DP\right\|}^{2}+{\left\|B-VP\right\|}^{2}+\alpha {\left\|G-VU\right\|}^{2}+\lambda_{1}{\left\|D\right\|}^{2}+\lambda_{2}{\left\|P\right\|}^{2}+\lambda_{3}{\left\|V\right\|}^{2}+\lambda_{4}{\left\|U\right\|}^{2},$$ (1)

where ||⋅|| is the Frobenius norm  .  The first three terms in Eq. 1 are used to obtain approximate decompositions of *A*, *B*, and *G*, respectively. The parameter *α* is used for controlling the effect of gene expression signatures on the model – note that setting *α* to 0 or 1 amounts to completely disregard or fully include expression data, respectively. The last four terms are used for regularization and their importance is controlled by parameters $\lambda_{i}$, for i=1,2,3,4. All learned matrices (D,V,P,U) are initialized with small random values with *D*, *V*, and *P* being nonnegative (see Methods).

### Evaluation

To evaluate the accuracy of our predictions, we used the DrugVirus.info database [7] that provides evidence of drug efficacy against viruses at different stages of drug development. We selected data about host-centric antivirals, resulting in a set of 435 known drug-virus associations at different stages of drug development between 79 drugs and 55 viruses. We used this dataset, that we shall refer to as “assessment set”, to evaluate the systems performance on the binary classification task of predicting whether a drug was effective or not for a specific virus.

Training our model amounts to learning appropriate drug and virus representations in latent space. This can be done using any available set of drug targets, virus host proteins and infection related gene expression profiles. In fact, our learning procedure does not make use of any information about drug efficacy against viruses – the data matrices we use for learning only encode the perturbations caused by drugs and viruses on the interactome and on the transcriptome. Importantly, using larger datasets for learning provides extra constraints to the system and therefore we expect to obtain better representations, which in turn should lead to more accurate predictions. In the experiments presented here, to learn drug and virus representations we used data from 2197 drugs approved by the US Drug and Food Administration (FDA) with known human targets from DrugBank [21,22], and 143 viruses that cause human diseases and have known host proteins from the Human-Virus Interaction Database (HDVIDB) [23] (see Methods for details about the datasets). Fifty-five out of these 143 viruses were also present in our assessment set, and therefore we could use the predictions obtained for them to assess the performance of our system. The accuracy of the predictions for the remaining 88 viruses could not be verified, but they are provided in S1 File. Finally, gene expression data were available for only 34 viruses, 25 of which were in our assessment set. The results presented here were obtained with the model that had the lowest value of the cost function (Eq. 1) among 200 models trained with different random initializations. The value of α was set to 1, thus we fully included of gene expression data.

**Fig 2:**
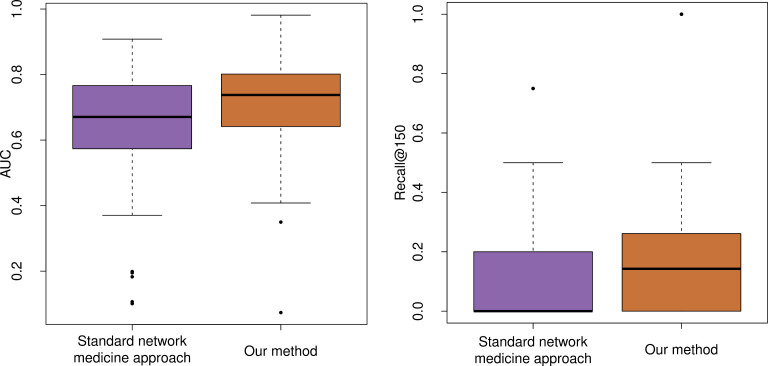
Evaluation of our method against standard network medicine approach. For each of the55 viruses in our assessment set, and each method (standard network medicine approach [5], and our method), we obtained a ranking of drugs according to their predicted efficacy score, and calculated the Area under the ROC curve (AUC) and Recall at top 150. Boxplots summarizing: left) the values of the area under the ROC curves for the different viruses; right) the recall at top 150 for the different viruses. For these experiments, the value of α (in Eq. 1) was set to 1, thereby indicating the inclusion of gene expression data. Our method achieved a median AUC of 0.73 and a median recall at the top 150 of 0.14, whereas the standard network medicine approach obtained a median AUC of 0.67 and a median recall of 0. Notably, the number of viruses with an AUC below 0.5 was 11 for the standard network medicine approach, but only 4 for our method.

For each of the 55 viruses we calculated the area under the Receiver Operating Characteristic (ROC) curve and the recall at top 150 (that is, the number of correct predictions in the top 150 out of the total number of drugs for that virus). These values are summarized in the boxplots in Fig 2, while Note A in S1 Text shows the performance for other levels of recall (at top 10,50,100,200). Fig 2 (and Note A in S1 Text) also reports, for comparison, the performance obtained using a standard network medicine approach based on diffusing the labels on the PPI network (for its implementation see Note B in S1 Text).

These results clearly demonstrate that our approach provides significantly greater accuracy than network medicine-based methods, without compromising applicability, as it can be applied to any compound with known targets and any virus with known host proteins. The good performance of our model is due to its ability to synergistically combine information about different viruses: the noise in the prediction obtained using network medicine-based approaches for one virus is reduced by integrating knowledge about other viruses. This can be easily verified by learning the drug and virus representations in latent space using only data about the 55 viruses in the assessment set, rather than the larger set of 143 viruses. Results of this experiments are presented in Note C in S1 Text where we can see that the performance of our system is lower when we use fewer viruses.

We found two approaches that are focused on host centric drugs, by Fiscon *et al* [16] (named SAveRUNNER) and by Li *et al* [24], but neither of the two are applicable to viruses in a general and systematic way. The first one requires the manual compilation of a list of diseases related to the viral disease in question, while the second one requires knowledge of virus host dependency genes. Although we realize it is a questionable choice, we applied the method of Fiscon *et al* [16] to our dataset by using the other viral diseases as potentially related (for method details see Note D in S1 Text). Results are reported in Note D in S1 Text. We also applied the approach of Li *et al* [24,25] – the only method we found that is focused on host centric drugs and does not rely on principles from network medicine – to the few viruses for which data is available (only 12 out of 55 viruses), and results are reported in Note E in S1 Text. Although these results may be somewhat influenced by the choice of related diseases in SaveRUNNER and by the limited number of viruses applicable in the case of Li et al., our method outperforms both approaches. Finally, other existing approaches for drug repurposing for viruses that are based on collaborative filtering [5,6] could not be used for comparisons, because they do not focus on host centric drugs.

### The effect of integrating gene expression data

One important aspect of our model is the possibility to include other types of data. In this paper we integrated transcriptomics information, but other types of data could be used in the same way by modifying Eq. 1. To test the effect of this data integration on the prediction we performed an ablation study, in which gene expression data was not used (this amounts to setting α=0 in Eq.1).

**Fig 3:**
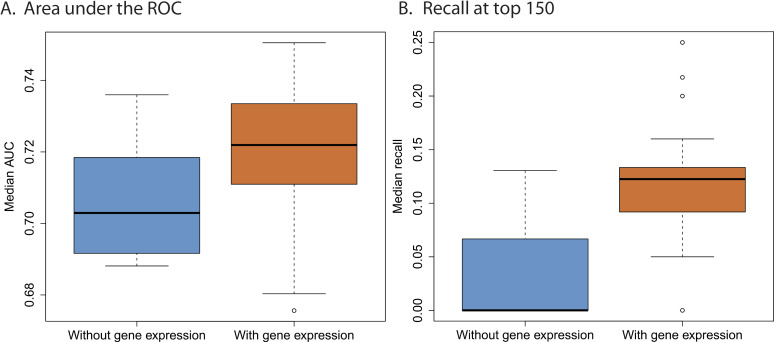
The effect of integrating gene expression data. We evaluated our method over 200 runs with and without gene expression data. **A) Area under the ROC curve.** For every run, we obtained the area under the ROC curve for each of the 55 viruses in our assessment set and calculated the median value. The 200 median values are summarized in the orange (with gene expression) and blue (without gene expression) boxplots. **B) Recall at top 150.** For every run, we calculated the median recall at top- 150 across 55 viruses. These values are summarized in the boxplots.

Fig 3 summarizes as boxplots the area under the ROC curve and the recall at top 150 when running our model with 200 different initializations. We observe that the integration of gene expression data improves results considerably. Making viruses closer in the latent space when they have similar gene expression profiles results in better predictions, because viruses with similar gene expression profiles will tend to respond similarly to a treatment. Notably, our model can exploit this information even when it is available for only a small subset of the viruses (as specified earlier, gene expression data was available only for 34 viruses out of 143).

### Biological interpretation

It is interesting to investigate whether the learned representations for drugs, viruses, proteins and genes encode meaningful information about the biology of the problem. Since each run of the method results in a different representation, we first needed to verify whether our representations are reproducible across different runs. We followed the reproducibility procedure used by Alexandrov et al. [26] to study cancer mutational signatures and by Galeano et al to study drug and side effects signatures [27] (see Methods). We saw that the average reproducibility scores were above 0.6, suggesting that the signatures were highly reproducible (see Note F in S1 Text). Therefore, any set of signatures could be considered for our analysis and the results presented here refer to the one with the lowest cost (Eq. 1) out of 200 different runs.

#### Drugs and viruses that share infection related pathways tend to have similar signatures.

Several studies suggest that drugs targeting pathways that are associated with a viral infection are likely to exhibit antiviral activity [8,10,13,28,29]. Thus, if our approach captures certain biological aspects of the problem, we expect drugs targeting pathways involved in a specific viral infection to display a signature resembling that of the virus. One way to check this is to test if drugs and viruses that share infection related metabolic pathways tend to have a higher signature similarity than those that do not.

We analysed KEGG (Kyoto Encyclopedia of Genes and Genomes) [30] pathways that were differentially expressed in human cell lines when treated with a drug [31] or infected by a virus in our dataset (see Methods). Any pathway divides drugs and viruses into two groups: those for which the pathway is differentially expressed, and those for which it is not. We wanted to verify whether there exist pathways such that the representation of drugs and viruses in the first group are closer than those in the second group. And, if such pathways exist, whether they were involved in a viral infection.

To do this, for each pathway, we collected two groups of similarities: one group containing cosine similarities between the representation of drugs and viruses that share the pathway, and one group containing cosine similarities between the representations of drugs and viruses that do not share the pathway. We then applied the one-sided Wilcoxon signed-rank test with multiple testing correction [32] to check for significant statistical differences between the two groups of similarities (see Methods).

After analysing the embedding similarities for each pathway, we found three pathways for which drugs and viruses are significantly closer when they share the same pathway. Importantly, all three pathways are linked to viral infection processes. The pathways were: “proteasome”, “regulation of actin cytoskeleton”, and “P53 signalling pathway”. The “proteasome” pathway is involved in host response to viral infections [33], “regulation of actin cytoskeleton” is manipulated by viruses during infection [34], and “P53 signalling pathway” have been linked to viral infections and potential treatments against viruses [35–37]. “Proteasome” is a protein-destroying apparatus involved in many essential cellular functions, including antigen processing for appropriate immune response and inflammatory responses [33]. The “regulation of the actin cytoskeleton” plays a crucial role in the control of cell shape and movement [38]. During a viral infection, it is often co-opted by the virus to facilitate the infection process [34], resulting in a reorganization of the actin cytoskeleton [39]. Finally, “p53 signalling pathway” has been shown to play a key role in antiviral innate immunity by both inducing apoptosis in response to viral infection, and enforcing the type I interferon response [37]. Because of this role, for evolutionary reasons, many viruses encode p53 antagonistic proteins [37].

**Fig 4:**
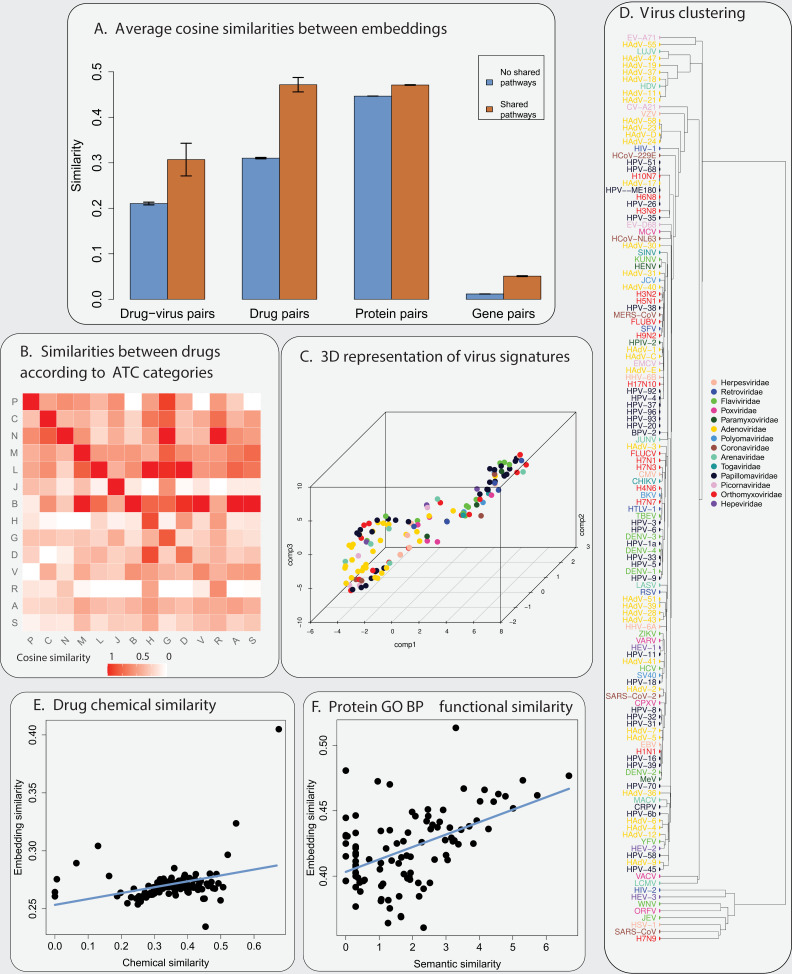
Relation between signatures and the biology of the problem. **A) Average cosine similarity between signatures.** For each type of pair between entities (drug-virus pairs, drug pairs, protein pairs and gene pairs), the blue and orange bars show the average cosine similarity of signatures for pairs that do not share pathways and pairs that share pathways, respectively. The pathways correspond to three selected KEGG pathways (“proteasome”, “regulation of actin cytoskeleton”, and “P53 signalling pathway”) for drug-virus pairs, all KEGG pathways for protein pairs and gene pairs, and DrugBank pathways for drug pairs. The black lines indicate the confidence interval around the means. Pairs that share pathways tend to have a higher similarity. **B) Similarities between drugs according to ATC categories**. In the heatmap, the value in position $\left(i,j\right)$ corresponds to the average cosine similarity of signatures between a drug that belongs to category *i* and a drug that belongs to category *j*. For visualization, we normalized all values according to the maximum value in the corresponding column. Drugs within the same category tend to have a more similar representation (see values on the diagonal). **C) 3D representation of virus signatures**. Dots correspond to virus signatures after embedding them in 3D using t-SNE. Colours correspond to virus families. **D) Virus clustering**. Dendrogram obtained by hierarchical clustering of the virus signatures using single linkage and cosine distance. Each colour corresponds to a virus family. Viruses belonging to some families, such as Adenoviridae, Papilomaviradae, and Flaviviridae, tend to form a few separate clusters. This is in accordance with the fact that the genomes of some viral families, such as Adenoviridae, form separate groups. **E) Drug chemical similarity.** Drug pairs were ordered according to the value of their chemical similarity and then divided into 100 groups of equal size. Each group is represented by a dot, whose x-coordinate is the average chemical similarity and y-coordinate is the average signature similarity between drugs within that group. We found a positive and significant correlation between signature similarity and drug chemical similarity (Spearman’s correlation coefficient = 0.326, *p-*value =0.0009). **F) Protein functional similarity**. Similarly, we divided the protein pairs into 100 groups according to their semantic similarity and obtained, for each group, the average signature similarity (*y*-axis) and the average semantic similarity (*x*-axis) according to the corresponding biological processes on Gene Ontology. We found a positive and significant correlation between signature similarity and functional similarity (Spearman’s correlation coefficient = 0.436, p-value = 5.918 $\times 10^{-6}$).

Fig 4a shows the average cosine similarities between drugs and virus signatures, for drug-virus pairs that do not share any of the three pathways (“proteasome”, “regulation of actin cytoskeleton”, and “P53 signalling pathway”) and for those that do. We observe that the second group has significantly higher similarities than the first one (p-value = $2.925\times 10^{-8}$).

#### Drug signatures are related to DrugBank pathways, ATC categories and chemical structures.

We investigated whether drugs with similar action and chemical structure tend to have a similar representation in the latent space. To test this, we analysed the relation between drug signatures, DrugBank drug-action pathways, ATC categories and drug chemical structure. We were able to collect drug information for different subsets of our original data: DrugBank pathways for 645 drugs, ATC categories for 1828 drugs, and chemical structure for 1800 drugs. We tested whether drug pairs that share drug pathways, ATC categories or have similar chemical structure are closer in the latent space than drug pairs that do not share these properties. As before, we used the cosine similarity to measure proximity between drugs pairs representations in the latent space.

We found that the cosine similarity of drug representations is higher for drug pairs that share DrugBank pathways than those that do not (Wilcoxon signed-rank test, *p-*value <$10^{-16}$), and higher for drug pairs within the same top-level category of ATC than those within different categories (Wilcoxon signed-rank test, *p-*value = $6.581\times 10^{-8}$) – see Fig 4a and 4b, respectively.

Each square in Fig 4b shows the mean similarity between embeddings of drugs within the corresponding ATC categories. In the Fig, each value was normalized by the highest values in its column (hence the matrix is not symmetric, and the main diagonal is not necessarily filled with ones). The normalization highlights that, in the embedding space, drugs are often closer to drugs that belong to the same ATC category than to drugs from different categories.

We also found that the cosine similarity between drug representations is positively correlated with chemical structure similarity (Spearman’s correlation coefficient = 0.326, *p-*value = 0.0009) – see Fig 4e. In the Fig, drug pairs were ordered according to the value of their chemical similarity and then divided into 100 groups of equal size. Each group is represented by a dot, whose x-coordinate is the average chemical similarity and y-coordinate is the average signature similarity between drugs within that group.

#### Drug target signatures are related to protein function.

We investigated whether drug target proteins that share similar functions tend to have similar representations in our latent space. To check this, we analysed the relation between drug target signatures and Gene Ontology (GO) terms. We were able to associate 1972, 2107, and 2106 drug target proteins to terms in the biological processes, cellular components, and molecular GO function categories, respectively (see Methods). We calculated the ResnikISM semantic similarities [40] between every pair of protein in each category and the cosine similarity between the corresponding drugs pairs representations in the latent space. Protein pairs were then ordered according to the value of their semantic similarity and then divided into 100 groups of equal size. Fig 4f (and Note G in S1 Text), shows each group as a dot, whose x-coordinate is the average semantic similarity and y-coordinate is the average signature similarity between proteins within that group. We measured the Spearman’s correlation coefficient (Scc) between these two similarities for all 100 points. We found a positive and significant correlation for all categories: biological processes (Scc = 0.436, *p-*”value = 5.918 $\times 10^{-6}$), cellular components (Scc = 0.332, *p-*value = 0.0007) and molecular function (Scc = 0.546, *p-*value = $4.093\times 10^{-9}$).

#### Protein and gene signatures are related to KEGG pathways.

We checked whether proteins that share metabolic pathways tend to have similar representations in our latent space. We were able to associate 4694 proteins in our original PPI set to KEEG pathways. We observed that protein pairs that share the same metabolic pathways tend to have a more similar representation than those that do not (Wilcoxon signed-rank test, *p-*value <$10^{-16}$) see Fig 4a.

We also checked whether genes that share metabolic pathways tend to have similar representations in our latent space. We were able to associate 4271 genes in our original set to KEGG metabolic pathways. We observed that gene pairs that share KEGG pathways have a more similar signature that those that do not (Wilcoxon signed-rank test, *p-*value <$10^{-16}$, see Fig 4a).

#### Analysis of the relation between virus signatures and virus families.

It is interesting to investigate whether virus signatures relate to the virus taxonomy. The small size of our dataset (143 viruses belonging to 14 viral families) does not warrant the use of statistical tests to compare virus families. Instead, we attempted to cluster and visually explore virus signatures across the different families: we performed a single-linkage hierarchical clustering of the virus signatures using cosine distance (one minus cosine similarity) (see Fig 4d) and plotted the signatures after embedding them in 3D using t-SNE (see Fig 4c).

The Figs show that representations of viruses belonging to some families, such as Adenoviridae, Papilomaviradae, and Flaviviridae, tend to cluster into a few separate groups. This is in accordance with the fact that the genomes of some viral families, such as Adenoviridae, form separate groups [41]. Interestingly, some dengue serotypes (DENV-1, DENV-3, and DENV-4) are represented close to each other in the dendrogram, suggesting that their representations capture their genetic similarity.

### Analysis of top predictions for Dengue and Ebola viruses

The aim of our method is to provide, for a given virus, a shortlist of the most promising drugs for repurposing. Our model outputs a matrix containing efficacy scores for each drug-virus pair, where each row represents a drug, and each column represents a virus. To rank the drugs for a specific virus, we focus on the corresponding column of the matrix and we sort it in descending order: a higher prediction score corresponds to a better rank position.

We stored the matrix of predictions obtained by our model on the aforementioned 143 viruses and 2,197 drugs in an Excel table (S1 File). To demonstrate the efficacy of our method, here we analyse a few of the top-ranked drugs in five columns of that table, corresponding to 4 serotypes of the Dengue virus (DENV-1, DENV-2, DENV-3, and DENV-4), and the Ebola virus (EBOV).

Among the top-ranked drugs for Dengue virus, we encountered Resveratrol, a phytoalexin secreted by plants. Resveratrol has demonstrated in vitro activity against Dengue virus due to its ability to inhibit HMGB1 migration out of the nucleus, thereby enhancing the production of interferon-stimulated genes [42]. Out of the 2,197 drugs in our dataset, Resveratrol was in the 9^th^ position for three serotypes of dengue virus (DENV-1, DENV-3, and DENV-4), and in the 11^th^ position for DENV-2.

Another drug that has shown in vitro activity against Dengue virus is Dasatinib [43,44], an anticancer agent. Its antiviral activity is attributed to the inhibition of Fyn kinase and c-Src protein kinase [43,44]. Dasatinib was encountered in position 17 for DENV-1 and DENV-4, position 18 for DENV-3, and position 21 for DENV-2.

Cyclosporine, an immunosuppressant agent, has demonstrated in-vitro activity against Dengue virus [45], possibly due to its ability to block the interaction between host cyclophilins and viral NS5 protein [45]. Cyclosporine was encountered in position 66 for DENV-4.

Among the top-ranked drugs for Ebola virus, we encountered Verapamil, a drug used to treat cardiovascular diseases that inhibited EBOV infection in vitro [46] and was shown to block endosomal calcium channels that are required by Ebola virus to entry into host cells. Our method ranked Verapamil in 7^th^ position, out of 2,197 drugs.

Estrogen receptor modulators, such as Raloxifene, Tamoxifen, and Diethylstilbestrol, also ranked highly in our model (15th, 23rd, and 48th positions, respectively) and have shown in vitro activity against EBOV [47]. In vivo experiments with other estrogen receptor modulators suggest that this drug class inhibits EBOV infection through mechanisms unrelated to the classical estrogen pathway [47].

The statin class of medications, primarily used to lower cholesterol levels, also achieved high predicted efficacy scores against EBOV, with Atorvastatin, Pitavastatin, Lovastatin, Rosuvastatin, and Simvastatin ranked between 31st and 65th positions. In vitro studies indicate that these drugs suppress EBOV infectivity by interfering with glycoprotein processing [48].

Anticancer drugs such as Sunitinib and Erlotinib achieved the 82nd and 91st highest prediction scores, respectively, and have demonstrated antiviral effects against EBOV in both in vitro and in vivo (murine model) studies [49]. In addition, clinical trials have been proposed to investigate their potential as EBOV infection treatments (ClinicalTrials.gov Identifier: NCT02380625) [50]. These drugs act by inhibiting host cell kinases, specifically AAK1 and GAK, which are essential for the intracellular trafficking of several viruses, including EBOV [49].

Other drugs that appear in the top-100 in our list and have demonstrated antiviral activity for Dengue virus and EBOV are shown in Note H in S1 Text.

## Discussion

Machine learning methods for drug repurposing rely on large datasets of drug-disease associations. Many of these methods are agnostic of the biology of the problem, which is framed simply as the problem of predicting known associations. For viruses, there are only a few approved antivirals, most of which are virus centric, directly targeting the pathogens.

To obtain predictions for a much larger set of drugs that could be repurposed for host centric therapies, we propose a method that exploits the biology of the problem. Our approach integrates different types of information about viral infections to learn latent space representations of viruses, drugs and genes that are biologically meaningful and represent biological processes involved in viral diseases and drug effects. This is done by effectively combining ideas from collaborative filtering and network medicine: we decompose matrices that are generated by diffusing processes on PPI networks. In this way, our method is able to synergistically combine information about network perturbations caused by different drugs and viruses, thus leading to better predictions.

From the machine learning point of view, an interesting aspect of our method is that it obtains predictions by multiplying latent representations obtained from different decompositions. Notice that this is different from standard matrix factorization techniques and also Collective Matrix Factorization [51], where predictions rely on the product of matrices obtained from the same decomposition. We have shown that, the larger the datasets involved in the decomposition the better the representations – this is because the system is more constrained. Better representations, in turn, lead to more accurate predictions.

We have shown that our method outperforms a standard network medicine approach, achieving higher AUC and recall. Notably, for 8 out of the 11 viruses that had an AUC of 0.5 or lower with the standard network medicine approach, our method produced an AUC greater than 0.5 (see Note I in S1 Text). Most of these viruses—LASV, HAdV-19, FLUBV, EMCV, HCoV-NL63, and EV-D68—are associated with less than 10 host proteins in our datasets. This suggests that the limited available data was insufficient for the standard network medicine approach to produce effective predictions. However, our method was able to overcome this limitation by integrating information across multiple viruses.

Our model is flexible, and it allows the integration of different types of data. We showcase this by integrating transcriptomics information of viral diseases. Our experiments show that integrating this data improves predictions, even when this data is available for only a small number of viruses.

We have shown that our method provides meaningful and reproducible representations for viruses, drugs, proteins and genes. Our signatures are related to drug chemical structure, protein function, and biological pathways involved in drug effects and viral infections. Thus, our learned representations might be useful for other tasks involving drugs, viruses, proteins or genes. These results also suggest that our latent features capture cellular mechanisms related to viral diseases and drug action.

One of the potential impacts of this work is to accelerate drug development for viral diseases. Our method can be applied to any virus, and it only requires the virus host proteins and drug target information. This data is becoming more readily available: Krogan *et al* [52] identified the 332 host proteins for SARS-CoV-2 only three months after the virus had been sequenced; large set of drug targets are currently known and can also be predicted with good accuracy [53,54].

This work aims to shortlist a set of drugs that could potentially be repurposed for a virus. Like other drug repurposing methods [2–5,15,16], our approach cannot predict dosage. Determining the appropriate dosage, along with evaluating drug’s safety and effectiveness against specific viruses, must be carried out experimentally in the later stages of drug development.

Like any machine learning algorithm, our method is affected by different sources of noise in the data, including incomplete knowledge of drug-target interactions, host proteins, protein interactions, and transcriptomics data. Additionally, approaches that diffuse information across large networks are sensitive to the number of seed nodes in the system. In our case, this means that drugs with a large number of known targets are likely to cause significant perturbations in the PPI network, resulting in high prediction scores. For example, drugs with many known targets, such as Fostamatinib, or certain vitamins and supplements, may receive disproportionately high efficacy scores. Therefore, the expertise and judgment of scientists are essential for filtering the model’s outputs before proceeding to in vitro validation.

All the predictions obtained by our model on the aforementioned 143 viruses are available in S1 File. When applying our model to new viruses, the first step is to construct new matrices B and G (in Fig 1) to be decomposed. We recommend including the largest possible number of viruses in these matrices, as the strong performance of our model stems from its ability to synergistically integrate information from different viruses. A suggested approach would be to combine the new viruses with those used in this study to create a more comprehensive dataset.

## Materials and methods

### Datasets

#### Interactome.

We used the PPI network obtained from Gysi et al [15]., which contains 327,924 interactions among 18,505 human proteins.

#### Drug-target associations.

We obtained FDA-approved drugs and their drug targets from DrugBank [21,22] and Gysi et al. [15] Our set of drugs consisted of 2197 FDA-approved drugs. Our set of drug target associations consisted of 14941 pairs of drug and targets.

#### Drug-virus associations.

We downloaded 1518 drug-virus associations from DrugVirus.info database [7]. To select host-centric antivirals, we filtered drugs that have human targets on the “Potential targets” field of DrugVirus.info database. We found 435 associations between 55 viruses that are available from HDVIDB [23] and have associations with 79 host-centric antivirals with known targets in the interactome. We refer to this set as “assessment set” and it is available in S2 File.

#### Host proteins.

We downloaded host protein data from HDVIDB [23]. We found 6152 host proteins for 143 viruses that cause human diseases with entries on the DrugVirus.info [7] database. For each virus, we considered the union of host proteins across different strains. Our final set of host proteins contains only those that have connections in the interactome. For mapping viruses between HDVIDB and DrugVirus.info databases, we used both virus names and abbreviations (see S3 File).

#### Virus families.

We obtained virus families from DrugVirus.info [7] database.

#### Gene expression signatures of viral diseases.

We downloaded gene expression data from Gene Expression Omnibus (GEO) [55] for 34 viruses. The GEO accession numbers, and further details are available in Note J in S1 Text.

#### DrugBank pathways.

We downloaded 877 pathways for 645 drugs from DrugBank [21,22].

#### KEGG pathways.

We downloaded 186 KEGG [30] pathways from MSigDB [33].

#### Differentially expressed pathways in cells treated with drugs.

We used DRUGPATH database [31] to obtain KEGG pathways that are significantly differently expressed in cell lines treated with drugs. We selected only pathways with significant *p-*value after multiple testing correction. After mapping drug names to drug bank IDs, we obtained 77 KEGG pathways that are differentially expressed for 548 drugs in our dataset.

#### Differentially expressed pathways in viral diseases.

We used all 186 KEGG metabolic pathways for the enrichment analysis of differentially expressed genes in 34 viral diseases. We used values in matrix *G* (see Section Inference of Input Matrices) for identifying genes that are up or down-regulated in viral infections. Then, for each virus, and each pathway, we performed a hypergeometric test for identifying pathways that are overrepresented in the set of down-regulated genes or in the set of up-regulated genes. We adjusted the p-values by the false discovery rate [32] and used a significance level of 5%.

### Construction of input matrices

The construction of input matrices *A* and *B* (Fig 1e) relies on the 2-step random walk kernel for measuring the level of perturbation caused by drugs and viruses, respectively. For each drug, the perturbation score corresponds to the maximum value of the 2-step random walk kernel between its targets and a given protein (Fig 1b). For a virus, the perturbation score corresponds to the maximum value of the 2-step random walk kernel between the host proteins and a given protein (Fig 1c). The construction of matrix *G* relies on tests of differential expression between infected cell lines (or tissues) and controls. For each virus, matrix *G* shows genes that are up or down-regulated (Fig 1d). In the following, we describe details on the 2-step random walk kernel and the construction of matrices *A*, *B* and *G*.

#### *p*-step random walk.

We represent the PPI network by a graph $G=\left(V,P\right)$, where $V=\left\{1,2,\ldots ,n_{P}\right\}$ is the set of nodes (proteins), and *E* is the set of links connecting the nodes (protein interactions). Let *W* be the adjacency matrix of *G*. Then, $W_{ij}=1$ if protein *i* and *j* interact and 0 otherwise. Let $\tilde{L}$ denote the normalized laplacian: $L=I–D^{-\frac{1}{2}}WD^{-\frac{1}{2}}$. Then the p-step random walk kernel [56] is defined as


$$K={\left(aI–L\right)}^{p},$$

where a≥2. We set *p* and *a* to 2. We used the implementation from diffuStats R package [57].

#### Matrix *A.*

Matrix *A* is a $n_{D}\times n_{P}$ matrix in which the entry $A_{ij}$ is 1 if drug *i* perturbates protein *j* according to a threshold $T_{1}$, and 0 otherwise. We obtained a perturbation score by diffusing drug targets on the PPI. For each drug *i* and protein *j* in the interactome, we define the perturbation score $p_{ij}$ as the maximum value of the 2-step random walk kernel between protein *j* and all targets of the drug *i*:


$$p_{ij}=\underset{t\text{in}T\left(i\right)}{\max}K_{tj},$$

where $T\left(i\right)$ is the set of targets of drug *i*, and *K* is the 2-step random walk kernel on the human interactome. We set the threshold $T_{1}$ as the 90% quantile of the perturbation scores. Then we defined *A* as:


$$A_{ij}= \{\begin{array}{c} 1,\text{if}p_{ij}\geq T_{1}\\ 0,\text{otherwise} \end{array}$$

We excluded each protein *j* in the interactome for which $p_{ij}<T_{1}$ for every drug *i*, that is, proteins that are not “reachable” from any drug through the diffusion of drug targets in the interactome. In addition, we removed proteins that are not “reachable” from any virus (see section below). This ensures that *A* and *B* will have the same columns. The resulting number of proteins was $n_{P}=17644$. We included data of $n_{D}=2197$ drugs.

#### Matrix *B.*

Matrix *B* is a $n_{V}\times n_{P}$ matrix in which the entry $B_{ij}$ is 1 if virus *i* perturbates protein *j* according to a threshold $T_{2}$, and 0 otherwise. We obtained a perturbation score by diffusing host proteins on the PPI. For each virus *i* and protein *j* in the interactome, we defined the perturbation score $p_{ij}$ as the maximum value of the 2-step random walk kernel between protein *j* and all host proteins of virus *i*:


$$p_{ij}=\underset{h\text{in}H\left(i\right)}{\max}K_{hj},$$

where $H\left(i\right)$ is the set of host proteins of virus *i*, and *K* is the 2-step random walk kernel on the human interactome. We set the threshold $T_{2}$ as the 90% quantile of the perturbation scores. Then we defined *B* as:


$$B_{ij}= \{\begin{array}{c} 1,\text{if}p_{ij}\geq T_{2}\\ 0,\text{otherwise} \end{array}$$

We excluded each protein *j* in the interactome for which $p_{ij}<T_{2}$ for every virus *i*, that is, proteins that are not “reachable” from any virus through the diffusion of host proteins in the interactome. In addition, we removed proteins that are not “reachable” from any drug, as explained in the section above. Thus, we ensure *B* will have the same columns as *A*.

#### Matrix *G.*

Matrix *G* is a $n_{V}\times n_{G}$ matrix describing gene expression profiles of viral infections. For 34 viruses, we downloaded gene expression data from human cell lines (or tissues) infected by the virus and control cell lines (tissues). Then, for each virus *i* and gene *j*, we obtained a z-score measuring the difference between the average expression in infected cell lines and control cell lines. To select the significant difference in expression we used a significance level of $10^{-6}$. We then defined *G* as


$$G_{ij}= \{\begin{array}{cc} 1, & \text{if gene}j\;\text{is significantly up}-\text{regulated for virus}i\\ -1, & \text{if gene}j\;\text{is significantly down}-\text{regulated for virus}i\\ 0, & \text{if gene}j\;\text{is not significantly up differentially expressed,}\\ & \text{or if data for gene}j\;\text{is not available for virus}i \end{array}$$

Notice that *G* has $n_{V}=143$ rows (viruses), but only 34 of them contain gene expression information. The number of columns is $n_{G}=17967$, which corresponds to the union of all annotated genes across the 34 gene expression profiles.

### Details of the learning algorithm

Our code is available at https://github.com/paccanarolab/VirusDrugRepo.

#### Initialization of the representations.

Our model learns non-negative matrices for representing drugs (*D*), viruses (*V*), and proteins (*P*), and a real valued matrix for representing genes (*U*). The gene representation may include negative values for capturing the different directions of changes in gene expression. We initialized *D*, *V*, and *P* with random values from a uniform distribution in the interval $\left[0,0.001\right]$. For initializing U, we used random values from a uniform distribution in the interval $\left[-0.001,0.001\right].$

#### Cost function optimization.

For optimizing the cost function in Eq. 1, we used Adam gradient [58]. We initialize the latent representations (*D*, *V*, *P*, and *U*) with random values (as described in the previous section), and at each iteration, we update their values according to the gradients and the second moments of the gradients. If the updates result in negative values for *D*, *V*, and *P* (that should be nonnegative), we simply replace these negative values by zeros [59]. The stopping criterion of the iterative process relies on the convergence criterion of the cost function. That is, our method stops when the difference between the cost function of the current iteration and the previous iteration is smaller than $10^{-4}$.

#### Hyperparameter setting.

We did not fine-tune any hyperparameter of our model. We set the number of hidden features to k=15. For controlling the contribution of gene expression data we used α=1. We set all regularization parameters to ${\text{λ}}_{1}={\text{λ}}_{2}={\text{λ}}_{3}={\text{λ}}_{4}=$0.1. Note that the performance of our method is not sensitive to the choices of the regularization parameters (see Note K in S1 Text).

### Similarity measures

#### Cosine similarity.

For measuring similarity between latent representations, we calculated the cosine similarity, that is defined as follows. Let *x* and *y* be *k* -dimensional vectors corresponding to representations learned by our model. Then the cosine similarity between *x* and *y* is obtained by the dot product of the vectors divided by the product of the norm of each vector:


$$\frac{xy^{T}}{\left\|x\right\|\left\|y\right\|},$$

where   denotes the Euclidian norm of a vector.

#### Chemical similarity.

To measure the chemical similarity between drugs, out of the 2197 drugs in our original set, we managed to obtain the SMILES representation for 1800 drugs from DrugBank [21,22]. Then, we obtained the Daylight fingerprint and calculated the Tanimoto similarity using python’s RDkit package (https://www.rdkit.org/).

#### Semantic similarity.

To calculate the functional semantic similarity, we used GOssTo [40,60]. We used the Resnik Integrated Similarity Metric (ISM)^40^ provided by GOssTo with default parameters. We restricted the GO relations to “is_a”, and “part_of”, and the evidence codes of the GO annotations to EXP, IDA, IPI, IMP, IGI, IEP, TAS, IC. We obtained biological process (BP) semantic similarities for 11223 proteins in our interactome, of which 1972 are also in our set of drug targets. For cellular components (CC), we obtained semantic similarity for 12734 proteins (2107 drug targets). Finally, we obtained molecular function (MF) semantic similarities for 14511 proteins (2106 drug targets).

### Statistical analysis

#### Statistical test of proximity in the latent space.

To test whether entity pairs in our model (e.g., drug-virus pairs, drug pairs, proteins pairs, virus pairs or gene pairs) are closer in the latent space than other entity pairs, we used the one-sided Wilcoxon signed-rank test between the corresponding cosine similarities of the representations. We considered a significance level of 0.05.

#### Correlation test.

To measure the correlation between the cosine similarities of representations learned by our model and other types of similarities (e.g., function similarity between proteins, chemical structural similarity between drugs), we used the Spearman’s correlation coefficient setting its significance level to 0.05.

#### Reproducibility analysis.

Following Alexandrov et al. [26], we evaluated the reproducibility of components by using these steps. First, we trained our model 200 times, each one with a different seed for generating pseudorandom numbers. Each independent run of the method gives a solution $\left\{D^{i},V^{i},P^{i},U^{i}\right\}$, where matrices $D^{i}$, $V^{i}$, $P^{i}$, $U^{i}$ denote representations for drugs, viruses, proteins, and genes, respectively. Then we selected 100 solutions $i_{1},i_{2},\ldots ,i_{100}$ with the lowest cost function at convergence (Eq. 1). We obtained matrices $\bar{D}$, $\bar{V}$, $\bar{P}$, $\bar{U}$ by concatenating the corresponding representations ($D^{i}$, $V^{i}$, $P^{i}$, $U^{i})$ across the 100 selected runs of the method. That is, matrix $\bar{D}$ corresponds to the concatenation of the columns of $D^{i_{1}}$, $D^{i_{2}}$,..., and $D^{i_{100}}$. Matrix $\bar{V}$ corresponds to the concatenation of the columns of $V^{i_{1}}$, $V^{i_{2}}$,..., and $V^{i_{100}}$. We obtained $\bar{P}$ by aggregating the rows of $P^{i_{1}}$, $P^{i_{2}}$,.., and $P^{i_{100}}$, and $\bar{U}$ by aggregating the rows of $U^{i_{1}}$, $U^{i_{1}}$,..., and $U^{i_{100}}$. For each concatenated matrix ($\bar{D}$, $\bar{V}$, $\bar{P}$, $\bar{U}$), we clustered the 100k aggregated features into *k* clusters, where k=15 (number of hidden features). For $\bar{D}$ and $\bar{V}$, it corresponds to clustering the columns, whereas for $\bar{P}$, and $\bar{U}$ it corresponds to clustering the rows. To obtain the clusters, we used kmeans++ based on cosine distance implemented on the GMKMcharlie R package [61]. As each run of kmeans results in a different clustering, we repeated the experiment 100 times and used a different seed for each run. For each matrix ($\bar{D}$, $\bar{V}$, $\bar{P}$, $\bar{U}$), we selected the clustering we highest cosine-similarity-based average silhouette width [62], which measures the tightness and separation of the clusters. We can interpret the silhouette width as a measure of reproducibility of each signature component. We show the silhouette for each of the k clusters if Fig S5, S6, S7, and S8 for $\bar{D}$, $\bar{V}$, $\bar{P}$, $\bar{U}$, respectively. The final reproducibility score is obtained by the average silhouette across all components.

## Supporting information

S1 Text Supporting text describing setup of experiments and additional analyses. It includes further evaluation of our method (recall at 20, 50, 100, and 200, top predictions for dengue and Ebola, AUC for specific viruses), setup of standard machine learning approach, additional experiments (effect of adding viruses, comparison against SaveRUNNER, and Li et al, reproducibility analysis, relation between drug target signature and functional similarity for cellular components and molecular function, and results with different hyperparameters), and gene expression data information.
(PDF)

S1 File Spreadsheet containing predictions obtained by our model for 143 viruses and 2917 FDA-approved drugs.
(XLSX)

S2 File Spreadsheet containing host centric drug-viruas associations from DrugVirus.info used for evaluating our approach.
(XLSX)

S3 File Spreadsheet mapping viruses between HDVIDB and DrugVirus.info databases.
(XLSX)

## References

[1] WHO. Coronavirus (COVID-19) Dashboard [Internet]. n.d. [cited 2023 Jun 20]. Available from: https://covid19.who.int

[2] Zhang W, Yue X, Lin W, Wu W, Liu R, Huang F, et al. Predicting drug-disease associations by using similarity constrained matrix factorization. BMC Bioinformatics. 2018;19(1):233. doi: 10.1186/s12859-018-2220-4 29914348 PMC6006580

[3] Yang M, Wu G, Zhao Q, Li Y, Wang J. Computational drug repositioning based on multi-similarities bilinear matrix factorization. Brief Bioinform. 2021;22(4):bbaa267. doi: 10.1093/bib/bbaa267 33147616

[4] Sadeghi S, Lu J, Ngom A. A network-based drug repurposing method via non-negative matrix factorization. Bioinformatics. 2022;38(5):1369–77. 34875000 10.1093/bioinformatics/btab826 PMC8825773

[5] Santos S de S, Torres M, Galeano D, Sánchez MDM, Cernuzzi L, Paccanaro A. Machine learning and network medicine approaches for drug repositioning for COVID-19. Patterns (N Y). 2022;3(1):100396. doi: 10.1016/j.patter.2021.100396 34778851 PMC8576113

[6] Tang X, Cai L, Meng Y, Xu J, Lu C, Yang J. Indicator regularized non-negative matrix factorization method-based drug repurposing for COVID-19. Frontiers in Immunology. 2020;11:603615. 33584672 10.3389/fimmu.2020.603615 PMC7878370

[7] Andersen PI, Ianevski A, Lysvand H, Vitkauskiene A, Oksenych V, Bjørås M, et al. Discovery and development of safe-in-man broad-spectrum antiviral agents. Int J Infect Dis. 2020;93:268–76. doi: 10.1016/j.ijid.2020.02.018 32081774 PMC7128205

[8] Zumla A, Rao M, Wallis RS, Kaufmann SHE, Rustomjee R, Mwaba P, et al. Host-directed therapies for infectious diseases: current status, recent progress, and future prospects. Lancet Infect Dis. 2016;1(4):e47-63. doi: 10.1016/S1473-3099(16)00078-5 27036359 PMC7164794

[9] Dwek RA, Bell JI, Feldmann M, Zitzmann N. Host-targeting oral antiviral drugs to prevent pandemics. Lancet. 2022;399(10333):1381–2. doi: 10.1016/S0140-6736(22)00454-8 35344736 PMC8956295

[10] Costa VV, Resende F, Melo EM, Teixeira MM. Resolution pharmacology and the treatment of infectious diseases. Br J Pharmacol. 2024;181(7):917–37. doi: 10.1111/bph.16323 38355144

[11] Santos PC, Teixeira MM, Souza DG. Opportunities for the development of novel therapies based on host-microbial interactions. Pharmacol Res. 2016;112:68–83. doi: 10.1016/j.phrs.2016.04.005 27107789

[12] Wallis RS, O’Garra A, Sher A, Wack A. Host-directed immunotherapy of viral and bacterial infections: past, present and future. Nat Rev Immunol. 2023;23(2):121–33. doi: 10.1038/s41577-022-00734-z 35672482 PMC9171745

[13] Mahajan S, Choudhary S, Kumar P, Tomar S. Antiviral strategies targeting host factors and mechanisms obliging ssRNA viral pathogens. Bioorganic & Medicinal Chemistry. 2021;46(9):116356. 34416512 10.1016/j.bmc.2021.116356 PMC8349405

[14] Kaufmann SHE, Dorhoi A, Hotchkiss RS, Bartenschlager R. Host-directed therapies for bacterial and viral infections. Nat Rev Drug Discov. 2018;17(1):35–56. doi: 10.1038/nrd.2017.162 28935918 PMC7097079

[15] Gysi D, Valle Í do, Zitnik M, Ameli A, Gan X, Varol O, et al. Network medicine framework for identifying drug-repurposing opportunities for COVID-19. Proceedings of the National Academy of Sciences. 2021;118(19):e2025581118. 10.1073/pnas.2025581118 PMC8126852 33906951

[16] Fiscon G, Conte F, Farina L, Paci P. SAveRUNNER: A network-based algorithm for drug repurposing and its application to COVID-19. PLoS Comput Biol. 2021;17(2):e1008686. doi: 10.1371/journal.pcbi.1008686 33544720 PMC7891752

[17] Guney E, Menche J, Vidal M, Barábasi A-L. Network-based in silico drug efficacy screening. Nat Commun. 2016;7:10331. doi: 10.1038/ncomms10331 26831545 PMC4740350

[18] Cáceres JJ, Paccanaro A. Disease gene prediction for molecularly uncharacterized diseases. PLoS Comput Biol. 2019;15(7):e1007078. doi: 10.1371/journal.pcbi.1007078 31276496 PMC6636748

[19] Barabási AL, Gulbahce N, Loscalzo J. Network medicine: a network-based approach to human disease. Nature Reviews Genetics. 2011;12(1):56–68. 10.1038/nrg2918 PMC3140052 21164525

[20] Lee DD, Seung HS. Learning the parts of objects by non-negative matrix factorization. Nature. 1999;401(6755):788–91. doi: 10.1038/44565 10548103

[21] (Knox C, Law V, Jewison T, Liu P, Ly S, Frolkis A, et al. DrugBank 3.0: a comprehensive resource for “omics” research on drugs. Nucleic Acids Res. 2011;39(suppl_1):D1035-41. doi: 10.1093/nar/gkq1126 21059682 PMC3013709

[22] (Wishart DS, Feunang YD, Guo AC, Lo EJ, Marcu A, Grant JR, et al. DrugBank 5.0: a major update to the DrugBank database for 2018. Nucleic Acids Res. 2018;46(D1):D1074–82. doi: 10.1093/nar/gkx1037 29126136 PMC5753335

[23] Yang X, Lian X, Fu C, Wuchty S, Yang S, Zhang Z. HVIDB: a comprehensive database for human–virus protein–protein interactions. Brief Bioinform. 2021;22(2):832–44. 33515030 10.1093/bib/bbaa425

[24] Li Z, Yao Y, Cheng X, Chen Q, Zhao W, Ma S, et al. A computational framework of host-based drug repositioning for broad-spectrum antivirals against RNA viruses. iScience. 2021;24(3):102148. doi: 10.1016/j.isci.2021.102148 33665567 PMC7900436

[25] Li Z, Yao Y, Cheng X, Li W, Fei T. An *in silico* drug repositioning workflow for host-based antivirals. STAR Protoc. 2021;2(3):100653. doi: 10.1016/j.xpro.2021.100653 34286288 PMC8273420

[26] Alexandrov LB, Nik-Zainal S, Wedge DC, Aparicio SAJR, Behjati S, Biankin AV, et al. Signatures of mutational processes in human cancer. Nature. 2013;500(7463):415–21. doi: 10.1038/nature12477 23945592 PMC3776390

[27] Galeano D, Li S, Gerstein M, Paccanaro A. Predicting the frequencies of drug side effects. Nature Communications. 2020;11(1):4575. doi: 10.1038/s41467-020-18312-5 PMC7486409 32917868

[28] Smith SB, Dampier W, Tozeren A, Brown JR, Magid-Slav M. Identification of common biological pathways and drug targets across multiple respiratory viruses based on human host gene expression analysis. PLoS One. 2012;7(3):e33174. doi: 10.1371/journal.pone.0033174 22432004 PMC3303816

[29] Tripathi D, Sodani M, Gupta PK, Kulkarni S. Host directed therapies: COVID-19 and beyond. Curr Res Pharmacol Drug Discov. 2021;2:100058. doi: 10.1016/j.crphar.2021.100058 34870156 PMC8464038

[30] Kanehisa M, Goto S. KEGG: kyoto encyclopedia of genes and genomes. Nucleic Acids Research. 2000;28(1):27–30. 10592173 10.1093/nar/28.1.27 PMC102409

[31] Jaundoo R, Craddock T. DRUGPATH: The Drug Gene Pathway Meta-Database. Int J Mol Sci. 2020;21(9):3171. 32365960 10.3390/ijms21093171 PMC7246871

[32] Benjamini Y, Hochberg Y. Controlling the false discovery rate: a practical and powerful approach to multiple testing. Journal of the Royal Statistical Society: Series B (Methodological). 1995;57(1):289–300. doi: 10.1111/j.2517-6161.1995.tb02031.x

[33] (Liberzon A, Subramanian A, Pinchback R, Thorvaldsdottir H, Tamayo P, Mesirov J. Molecular signatures database (MSigDB) 3.0. Bioinformatics. 2011;27(12):1739–40. 21546393 10.1093/bioinformatics/btr260 PMC3106198

[34] Cudmore S, Reckmann I, Way M. Viral manipulations of the actin cytoskeleton. Trends Microbiol. 1997;5(4):142–8. doi: 10.1016/S0966-842X(97)01011-1 9141188 10.1016/S0966-842X(97)01011-1

[35] Fantacuzzi M, Amoroso R, Ammazzalorso A. PPAR Ligands Induce Antiviral Effects Targeting Perturbed Lipid Metabolism during SARS-CoV-2, HCV, and HCMV Infection. Biology (Basel). 2022;11(1):114. doi: 10.3390/biology11010114 35053112 PMC8772958

[36] Layrolle P, Payoux P, Chavanas S. Ppar gamma and viral infections of the brain. International Journal of Molecular Sciences. 2021;22(16):8876. 34445581 10.3390/ijms22168876 PMC8396218

[37] Rivas C, Aaronson SA, Munoz-Fontela C. Dual Role of p53 in Innate Antiviral Immunity. Viruses. 2010;2(1):298–313. doi: 10.3390/v2010298 21994612 PMC3185551

[38] Villalonga P, Ridley AJ. Rho GTPases and Actin Cytoskeleton Dynamics. In: Lennarz WJ, Lane MD, editors. Encyclopedia of Biological Chemistry (Second Edition) [Internet]. Waltham: Academic Press; 2013 [cited 2024 Feb 11], 111–5. Available from: https://www.sciencedirect.com/science/article/pii/B9780123786302004394

[39] Taylor MP, Koyuncu OO, Enquist LW. Subversion of the actin cytoskeleton during viral infection. Nat Rev Microbiol. 2011;9(6):427–39. doi: 10.1038/nrmicro2574 21522191 PMC3229036

[40] Yang H, Nepusz T, Paccanaro A. Improving GO semantic similarity measures by exploring the ontology beneath the terms and modelling uncertainty. Bioinformatics. 2012;28(10):1383–9. 22522134 10.1093/bioinformatics/bts129

[41] Akello JO, Kamgang R, Barbani MT, Suter-Riniker F, Aebi C, Beuret C, et al. Genomic analyses of human adenoviruses unravel novel recombinant genotypes associated with severe infections in pediatric patients. Sci Rep. 2021;11(1):24038. doi: 10.1038/s41598-021-03445-y 34912023 PMC8674331

[42] Zainal N, Chang C, Cheng Y, Wu Y, Anderson R, Wan S, et al. Resveratrol treatment reveals a novel role for HMGB1 in regulation of the type 1 interferon response in dengue virus infection. Scientific Reports. 2017;7:42998. doi: 10.1038/s41598-017-04358-5 28216632 PMC5316936

[43] de Wispelaere M, LaCroix AJ, Yang PL. The small molecules AZD0530 and dasatinib inhibit dengue virus RNA replication via Fyn kinase. J Virol. 2013;87(13):7367–81. doi: 10.1128/JVI.00632-13 23616652 PMC3700292

[44] Chu J, Yang P. c-Src protein kinase inhibitors block assembly and maturation of dengue virus. Proceedings of the National Academy of Sciences of the United States of America. 2007;104(9):3520–5. 17360676 10.1073/pnas.0611681104 PMC1805510

[45] Qing M, Yang F, Zhang B, Zou G, Robida JM, Yuan Z, et al. Cyclosporine inhibits flavivirus replication through blocking the interaction between host cyclophilins and viral NS5 protein. Antimicrob Agents Chemother. 2009;53(8):3226–35. doi: 10.1128/AAC.00189-09 19451286 PMC2715601

[46] Sakurai Y, Kolokoltsov AA, Chen C-C, Tidwell MW, Bauta WE, Klugbauer N, et al. Ebola virus. Two-pore channels control Ebola virus host cell entry and are drug targets for disease treatment. Science. 2015;347(6225):995–8. doi: 10.1126/science.1258758 25722412 PMC4550587

[47] Johansen L, Brannan J, Delos S, Shoemaker C, Stossel A, Lear C, et al. FDA-approved selective estrogen receptor modulators inhibit Ebola virus infection. Science Translational Medicine. 2013;5(190):190ra79. 10.1126/scitranslmed.3005471 PMC3955358 23785035

[48] Shrivastava-Ranjan P, Flint M, Bergeron É, McElroy A, Chatterjee P, Albariño C. Statins suppress Ebola virus infectivity by interfering with glycoprotein processing. mBio. 2018;9(3):e00660-18. doi: 10.1128/mBio.00660-18 29717011 PMC5930306

[49] Bekerman E, Neveu G, Shulla A, Brannan J, Pu S, Wang S. Anticancer kinase inhibitors impair intracellular viral trafficking and exert broad-spectrum antiviral effects. J Clin Invest. 2017;127(4):1338–52. 28240606 10.1172/JCI89857 PMC5373883

[50] Berry SM, Petzold EA, Dull P, Thielman NM, Cunningham CK, Corey GR, et al. A response adaptive randomization platform trial for efficient evaluation of Ebola virus treatments: A model for pandemic response. Clin Trials. 2016;13(1):22–30. doi: 10.1177/1740774515621721 26768569 PMC5583707

[51] Singh AP, Gordon GJ. Relational learning via collective matrix factorization. In: Proceedings of the 14th ACM SIGKDD international conference on Knowledge discovery and data mining [Internet]. New York, NY, USA: Association for Computing Machinery; 2008 [cited 2023 Mar 28], 650–8. (KDD’08). Available from: doi: 10.1145/1401890.1401969

[52] Gordon DE, Jang GM, Bouhaddou M, Xu J, Obernier K, White KM, et al. A SARS-CoV-2 protein interaction map reveals targets for drug repurposing. Nature. 2020;583(7816):459–68. doi: 10.1038/s41586-020-2286-9 32353859 PMC7431030

[53] Luo Y, Zhao X, Zhou J, Yang J, Zhang Y, Kuang W, et al. A network integration approach for drug-target interaction prediction and computational drug repositioning from heterogeneous information. Nat Commun. 2017;8(1):573. doi: 10.1038/s41467-017-00680-8 28924171 PMC5603535

[54] Ye Q, Hsieh C-Y, Yang Z, Kang Y, Chen J, Cao D, et al. A unified drug-target interaction prediction framework based on knowledge graph and recommendation system. Nat Commun. 2021;12(1):6775. doi: 10.1038/s41467-021-27137-3 34811351 PMC8635420

[55] Edgar R, Domrachev M, Lash AE. Gene expression omnibus: NCBI gene expression and hybridization array data repository. Nucleic Acids Research. 2002;30(1):207–10. doi: 10.1093/nar/30.1.207 11752295 PMC99122

[56] Smola AJ, Kondor R. Kernels and Regularization on Graphs. In: Schölkopf B, Warmuth MK, editors. Learning Theory and Kernel Machines. Berlin, Heidelberg: Springer; 2003, 144–58. (Lecture Notes in Computer Science).

[57] Picart-Armada S, Thompson W, Buil A, Perera-Lluna A. diffuStats: an R package to compute diffusion-based scores on biological networks. Bioinformatics. 2018;34(3):533–4. 29029016 10.1093/bioinformatics/btx632 PMC5860365

[58] Diederik P, Ba J. Adam: a method for stochastic optimization. 2015.

[59] Berry MW, Browne M, Langville AN, Pauca VP, Plemmons RJ. Algorithms and applications for approximate nonnegative matrix factorization. Computational Statistics & Data Analysis. 2007;52(1):155–73. doi: 10.1016/j.csda.2006.11.006

[60] Caniza H, Romero A, Heron S, Yang H, Devoto A, Frasca M. GOssTo: a stand-alone application and a web tool for calculating semantic similarities on the Gene Ontology. Bioinformatics. 2014;30(15):2235–6. 24659104 10.1093/bioinformatics/btu144 PMC4103586

[61] Liu CW. GMKMcharlie: Unsupervised Gaussian Mixture and Minkowski and Spherical K-Means with Constraints [Internet]. 2021 [cited 2023 Mar 28]. Available from: https://CRAN.R-project.org/package=GMKMcharlie

[62] Rousseeuw P. Silhouettes: A graphical aid to the interpretation and validation of cluster analysis. J Comput Appl Math. 1987;20(1):53–65.

