# Supplementary material for: Host centric drug repurposing for viral diseases
Source: PLoS Comput Biol. 2025 Apr 2;21(4):e1012876. doi: 10.1371/journal.pcbi.1012876 (PMC12052139; doi:10.1371/journal.pcbi.1012876)
Supplement: S1 Text — It includes further evaluation of our method (recall at 20, 50, 100, and 200, top predictions for dengue and Ebola, AUC for specific viruses), setup of standard machine learning approach, additional experiments (effect of adding viruses, comparison against SaveRUNNER, and Li et al, reproducibility analysis, relation between drug target signature and functional similarity for cellular components and molecular function, and results with different hyperparameters), and gene expression data information. (PDF) [file pcbi.1012876.s001.pdf]

Supplementary Notes

Host centric drug repurposing for viral diseases

Suzana de Siqueira Santos[1], Haixuan Yang[2], Aldo Galeano [1], Alberto Paccanaro[1,3]

1: Escola de Matemática Aplicada, Fundação Getúlio Vargas, Rio de Janeiro, Brazil
2: School of Mathematical & Statistical Sciences, University of Galway, Galway, Ireland
3: Department of Computer Science, Centre for Systems and Synthetic Biology, Royal Holloway, University of London, Egham Hill, Egham, UK

Table of Contents

Note A. Recall at 20, 50, 100, and 200 3
Note B. Setup of our standard network medicine approach 4
Note C. Effect of adding viruses 5
Note D. Comparison against SaveRUNNER (Fiscon *et al*) 6
Note E. Comparison against Li et al 7
Note F. Reproducibility analysis 9
Note G. Relation between drug target signature and functional similarity for cellular components and molecular function GO categories 14
Note H. Top predictions for dengue and ebola viruses with in-vitro or in-vivo antiviral activity 15
Note I. AUC for viruses LASV, HAdV-2, MARV, HAdV-19, FLUBV, EMCV, HCoV-NL63, and EV-D68 18
Note J. Gene expression data 19
Note K. Results for different values of $\lambda 1, \lambda 2, \lambda 3, \lambda 4$ 22
References 24

Note A. Recall at 20, 50, 100, and 200

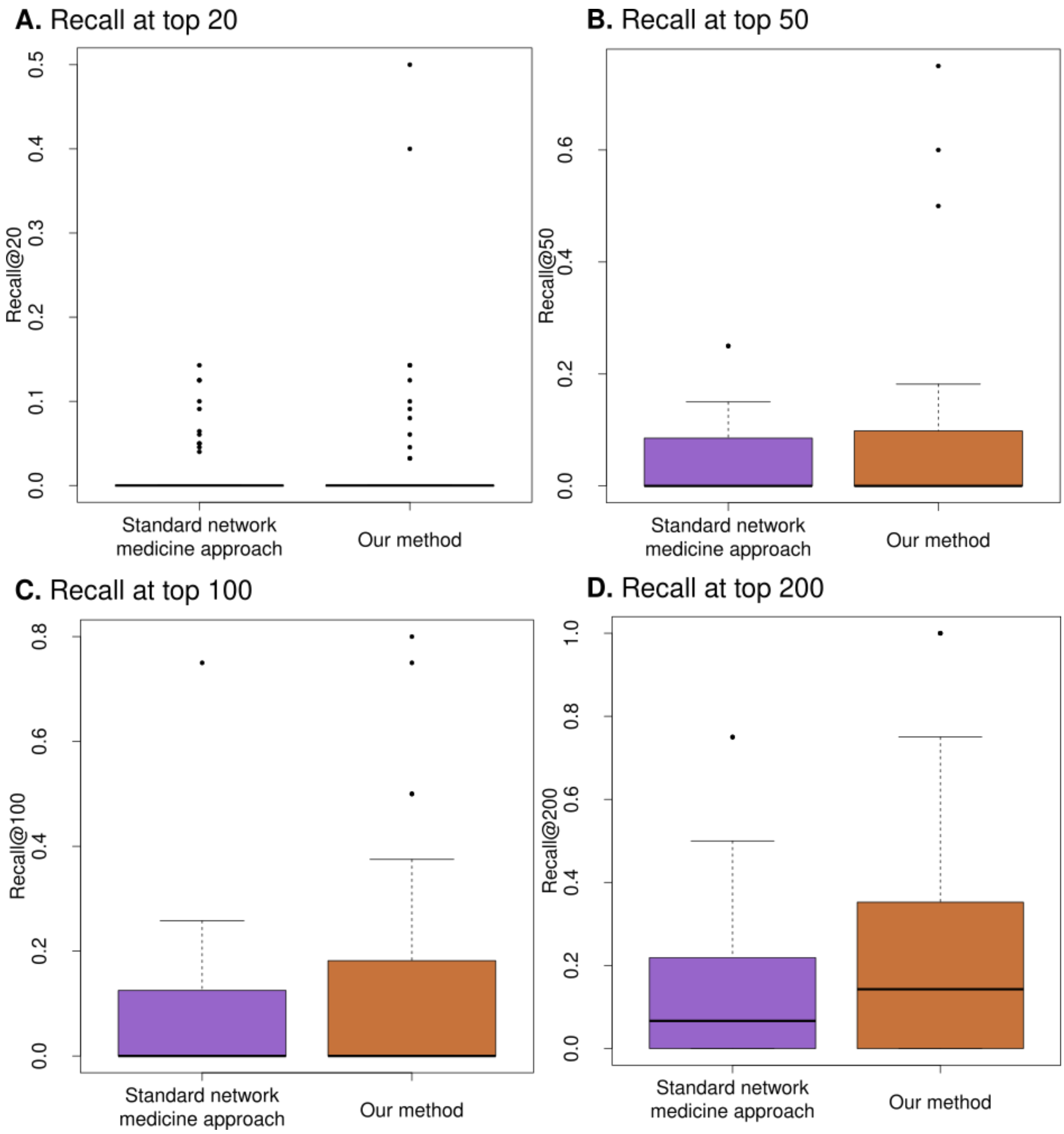

Fig A. *Recall at different top Ns. For each virus, we obtained the recall at top N, that is, the number of correct predictions on the top N, relatively to the total number of drugs with evidence against the virus. Each boxplot shows the recall for 55 viruses in our assessment set. We also report the performance of a standard network medicine approach for comparison (purple). Panels A, B, C, and D show the recalls at top 20, 50, 100, and 200, respectively.*

Note B. Setup of our standard network medicine approach

The standard network medicine approach that we used in Figure 2 relies on the diffusion of drug targets through the interactome. Our approach is similar to the one used by Santos et al[5] and it was chosen among a few similar approaches[15,17], because it had shown better performance[5].The idea is to measure how much host proteins are "perturbed" by drug targets. Given a virus, it obtains rankings of drugs based on five different diffusion kernels (p-step random walk, diffusion process, regularized laplacian, commute time, and inverse cosine)[56]. The output of the diffusion kernel is a square matrix K, where position K_{ij} indicates the proximity/similarity between the proteins i and j in the interactome. Each diffusion kernel relies on the normalized laplacian matrix obtained from the interactome. For each drug, the predition score is the sum of all kernel similarities between its targets and the host proteins of the virus of interest. The final ranking is the average ranking across all the diffusion kernels. To obtain the diffusion kernels, we used diffuStats R package[57], which was also used by Santos et al[5]. We used the same parameters as Santos et al. That is, the parameter p of p-Step random walk kernel was set to 2, and all other parameters were set to the default value in diffuStats. We run the algorithm using the same interactome, drug-targets associations and virus-host protein associations as used by our approach (see Methods).

Note C. Effect of adding viruses

To test whether adding viruses to our model can improve predictions, we compared the performance of our model containing 143 viruses (original model) against a version of our model containing only a subset of 55 viruses (assessment set). This subset corresponds to viruses that have associations with host centric antivirals in DrugVirus.info database, that is, all viruses for which we can assess the performance.

Our model relies on matrices A, B, and G of dimensions $n_D \times n_P$, $n_V \times n_P$, and $n_V \times n_G$, respectively. For the original model, we have $n_P = 17644$ proteins, and $n_V = 143$. For the smaller model, we have $n_P = 16891$ proteins, and $n_V = 55$ viruses. For both versions of the model, we have $n_D = 2197$ drugs, and $n_G = 17967$ genes.

We ran both versions of our model with 200 different random initializations, and evaluated the performance on the assessment set. For each run of the model, we obtained the median AUC and median recall at top 150 across the 55 viruses considered for evaluation. Fig B-A and Fig B-B show, respectively, the median AUC and median recall at top 150 for the two versions of the model. The AUC and recall are significantly higher in the original version of the model compared to the version that uses only the viruses in the assessment set (Wilcoxon signed rank tests, p-value < 10^{-16} for both tests). This suggests that adding extra viruses to our model can improve predictions.

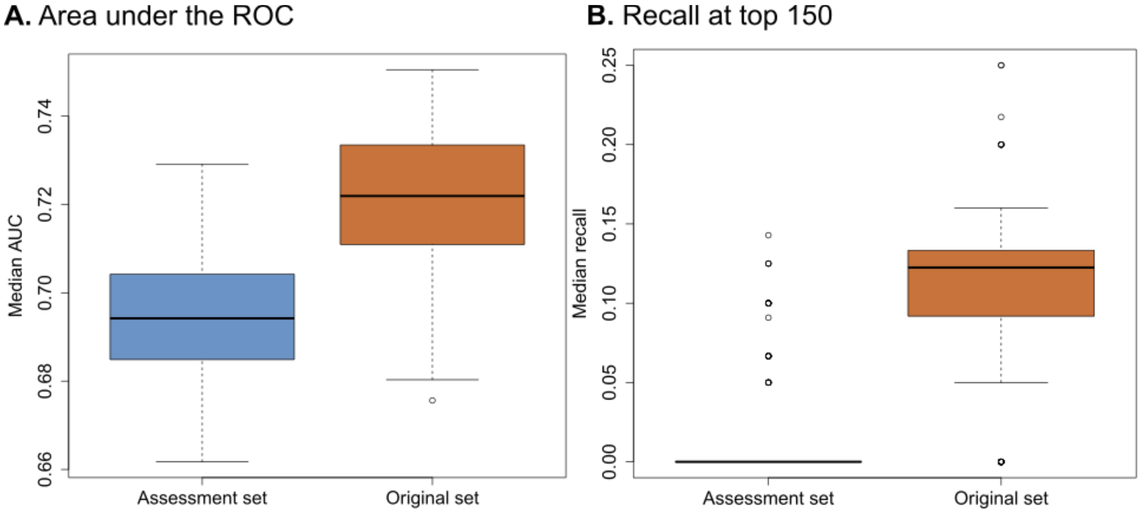

***Fig B**. Effect of adding viruses. We evaluated 200 runs of two versions of our method: one using the original set of 143 viruses, and another one that uses only 55 viruses in the assessment set. For both versions, we evaluated the performance on all viruses on the assessment set. For each run of the method and each virus, we obtained a ranking of drugs according to their predicted efficacy. A) Area under the ROC. For each run of the method, we obtained the median area under the ROC curve (AUC) across the 55 viruses considered for evaluation. The blue and orange boxplots show the median AUC for 200 runs of our method using the assessment set, and the original set of viruses, respectively. B) Recall at top 150. We obtained the median recall at top 150 across 55 viruses for each run of the method. The boxplots show the median recall for experiments relying on the assessment set (blue) and the original set of viruses (orange). We found that the model that relies on information from the largest set of viruses (original set) have the highest recall.*

Note D. Comparison against SaveRUNNER (Fiscon *et al*)

SAVERunner[16] builds a drug-disease network based on the distances between drug targets and disease modules. It was originally applied to COVID-19 and it requires a list of diseases related to the disease of interest, which is usually manually curated by a researcher. This data is in general not available. Therefore, to run the method on our dataset, given a query viral disease, we decided to use all the other viral diseases in the set as the diseases "related" to our query disease. While this choice is debatable as many viral infections are not necessarily related to each other, doing so allowed us to systematically compare SaveRUNNER with our approach.

Given a disease, SAveRUNNER computes the shortest path length between drug targets and the disease module (host protein module for viral diseases) on the interactome. Then it adds a weighted link between the drug and disease if the distance is significantly small according to a permutation-based test. Finally, SAveRUNNER clusters diseases and drugs into groups of densely connected nodes. Drugs and diseases that are within the same cluster have an increment on the prediction score. To run SAveRUNNER, we downloaded the R code from https://github.com/giuliafiscon/SAveRUNNER and used the default parameters.

Fig C shows a comparison of the AUC and recall for the 55 viruses in our assessment set, between our method, and SaveRUNNER.

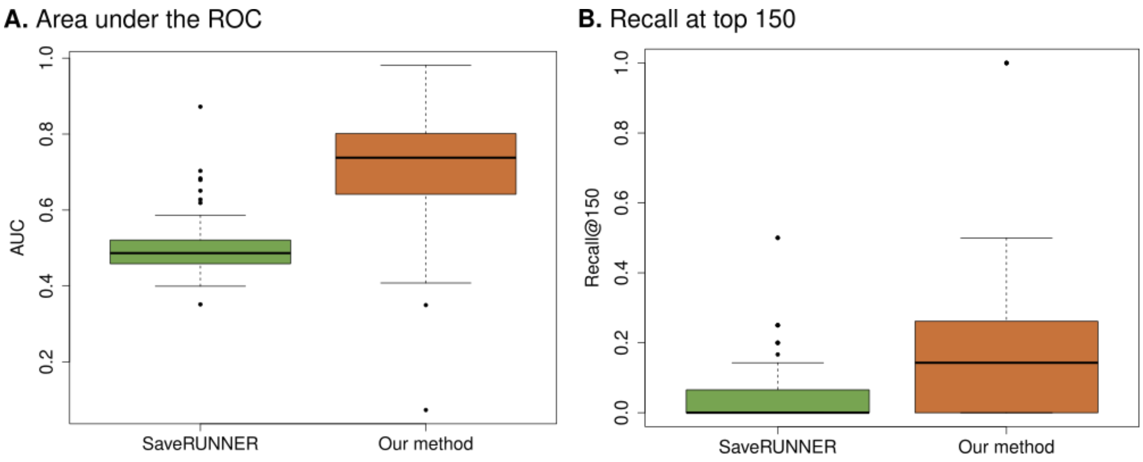

***Fig C**. Comparison against SaveRUNNER. For each of the 55 viruses in our assessment set, and each method (SaveRunner[16], and our method), we obtained a ranking of drugs according to their predicted efficacy score, and calculated the Area under the ROC curve and Recall at top 150. Boxplots summarizing: left) the values of the area under the ROC curves for the different viruses; right) the recall at top 150 for the different viruses.*

Note E. Comparison against Li et al

We also compared our method against the approach by Li et al[24]. They rely on drug target prediction for host dependency genes, that is, genes that are essential for a viral infection. We then followed their general pipeline[25], for a comparative analysis with our method.

To carry out DeepCPI, one of the main steps of the pipeline[24], we used InChI IDs available from their supplementary data. We found this information for 1880 out of 2197 drugs from our set of drugs.

Another requirement of the pipeline[25] is a list of host dependency genes, defined according to the criteria of Li et al[24]. Because they obtained host dependency genes only for Flaviridae virus family, SARS-CoV-2 virus, and other viruses from Coronaviridae family, we can only carry out the pipeline for these viruses.

For a comparative analysis, we selected viruses from our dataset that belong to available families. We show in Table A the relation of selected viruses, the corresponding families, and the group of host dependency genes. Notice that because Li et al[24] grouped the genes by family (except for SARS-CoV-2), the predictions by their approach are the same for viruses from the same family (except for SARS-CoV-2).

Table A. Intersection of viruses considered by our approach and by Li et al grouped by their set of host dependence genes (HDG) according to their corresponding families.

Group of HDGs	Virus	Family
Group 1	HCV	Flaviridae
	DENV-2	
	ZIKV	
	YFV	
	DENV-4	
	WNV	
	DENV-1	
	DENV-3	
Group 2	HCoV-229E	Coronaviridae
	MERS-CoV	
	SARS-CoV	
Group 3	SARS-CoV-2	Coronaviridae

For evaluating the methods, we used the same data described in Evaluation, in the main paper. We obtained the area under the ROC curve and the recall at top 150 for each virus, as shown in Fig D. Our results suggest that our method had better performance than Li et al.

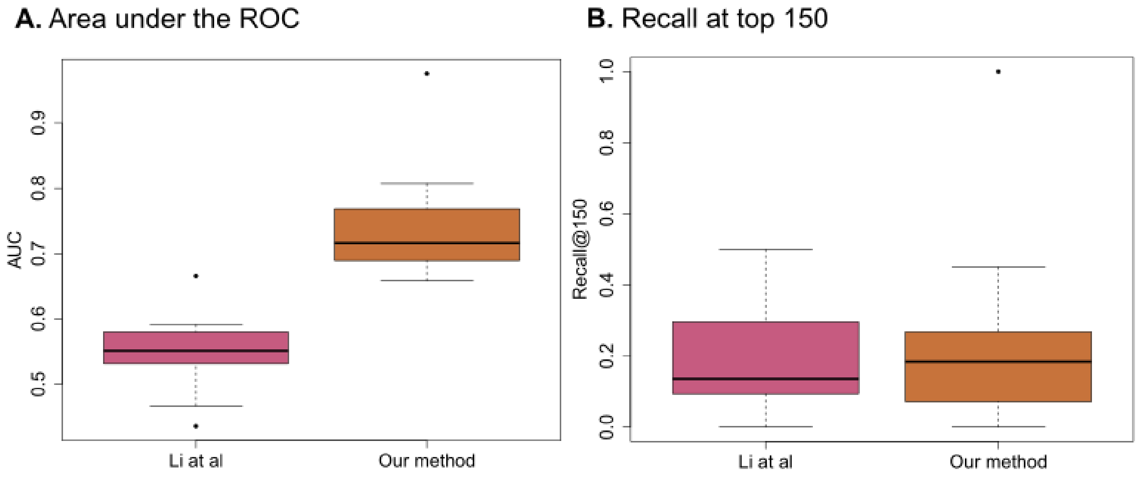

Fig D. *Evaluation of our method and the method of Li et al on a subset of 12 viruses. For each of the 12 viruses, we obtained a ranking of drugs according to their predicted efficacy score. A) Area under the ROC curve. For each method, we show a boxplot corresponding to the area under the ROC curve across the viruses. B) Recall at top 150. For each virus, we obtained the recall at top 150, that is, the number of correct predictions on the top relatively to the total number of drugs with evidence against the virus. Each boxplot shows the recall for 12 viruses available from Li et al. In both panels (A and B), our method had higher performance than Li et al.*

Note F. Reproducibility analysis

For analyzing the reproducibility of the components, we ran our method with 200 different seeds and selected the 100 runs with lowest cost function. If the components are reproducible, we expect that components will be similar across the different runs.

To verify that, we clustered all components obtained in the100 runs of the method into k=15 clusters. We expect that each cluster will correspond to a hidden feature in our model. Then, if the components are reproducible, we would identify 15 well separated clusters of approximately 100 components.

For measuring the reproducibility of the components obtained by our model, we obtained the silhouette statistic. The closer to 1 the silhouette statistic is, the better the clustering is, and the more reproducible the components are.

Fig E, F, G, and H show the silhouette statistic of each cluster for drugs, viruses, proteins, and genes, respectively.

In each plot, the consecutive bars of same color indicate the silhouette statistic of components within the same cluster. For each cluster, the three numbers on the right indicate the cluster ID, the cluster size, and the average silhouette of the cluster, respectively.

For drugs, viruses, proteins, and genes, we obtained a reproducibility score (average silhouette across all the clusters) of 0.95, 0.66, 0.94, and 0.81, respectively. Most of clusters have a size of approximately 100, as expected.

Our results suggest that our representations are highly reproducible with a reproducibility score higher than 60%.

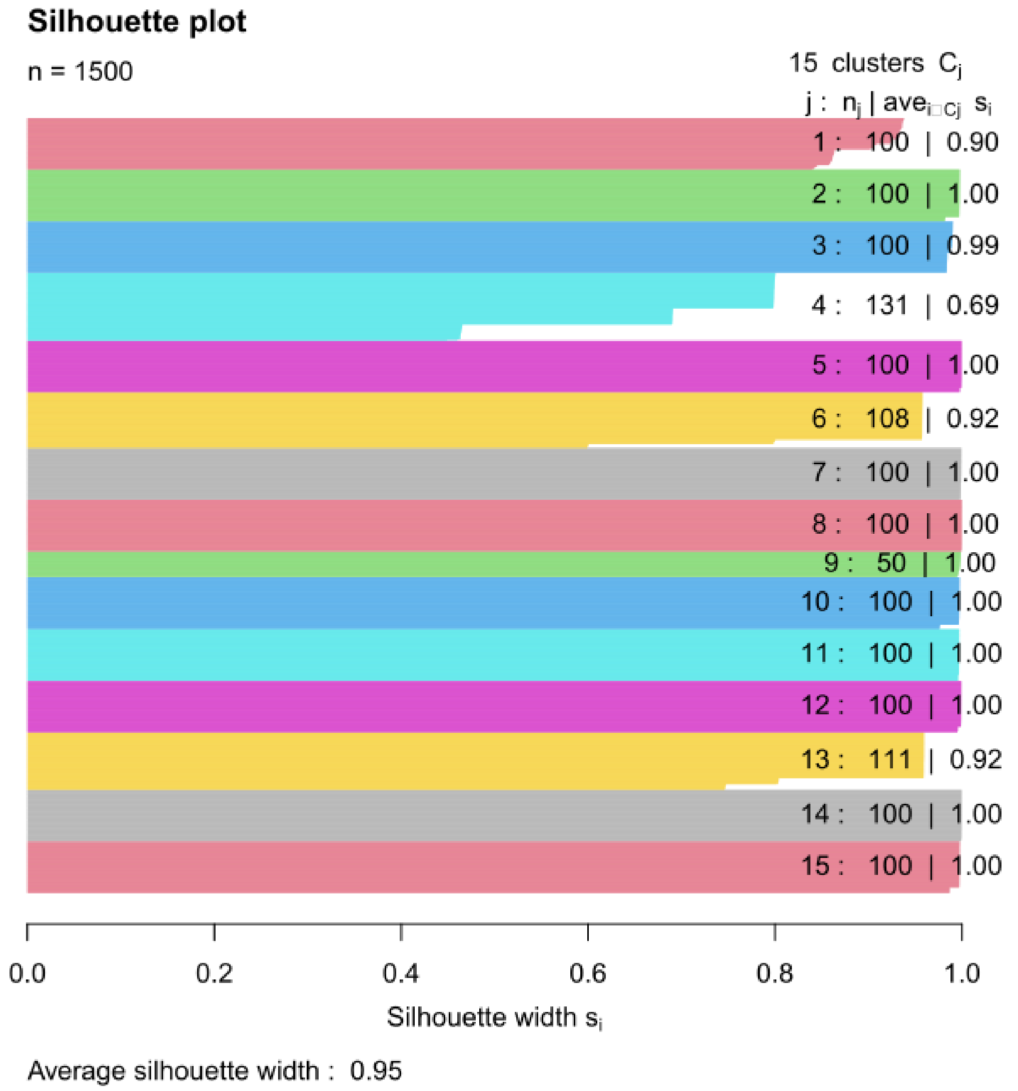

Fig E*. Reproducibility of components for drug representation. The consecutive bars of same color indicate the silhouette statistic of components within the same cluster. For each cluster, the numbers on the right indicate the cluster id, the cluster size and the average silhouette. The average silhouette among all the clusters (reproducibility score) is shown on the bottom.*

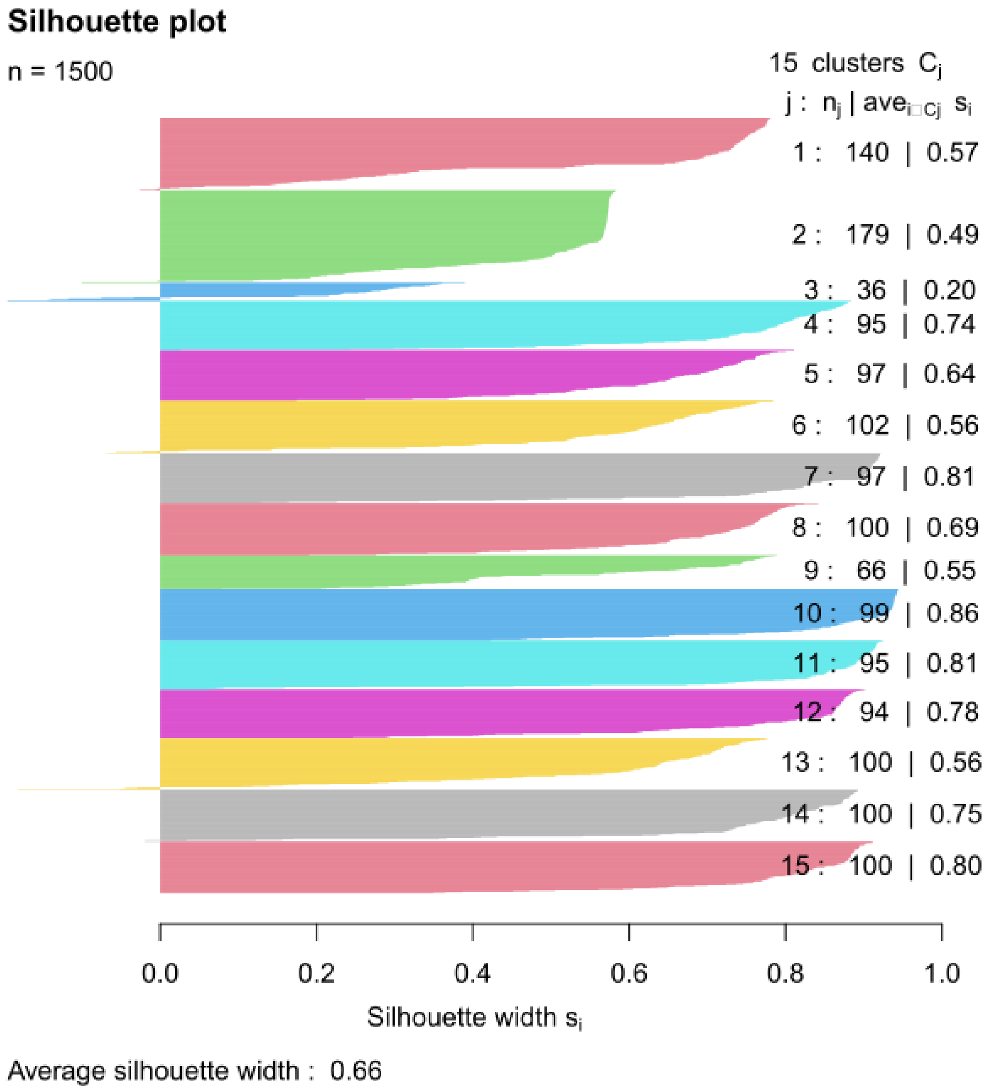

Fig F. *Reproducibility of components for virus representation. The consecutive bars of same color indicate the silhouette statistic of components within the same cluster. For each cluster, the numbers on the right indicate the cluster id, the cluster size and the average silhouette. The average silhouette among all the clusters (reproducibility score) is shown on the bottom.*

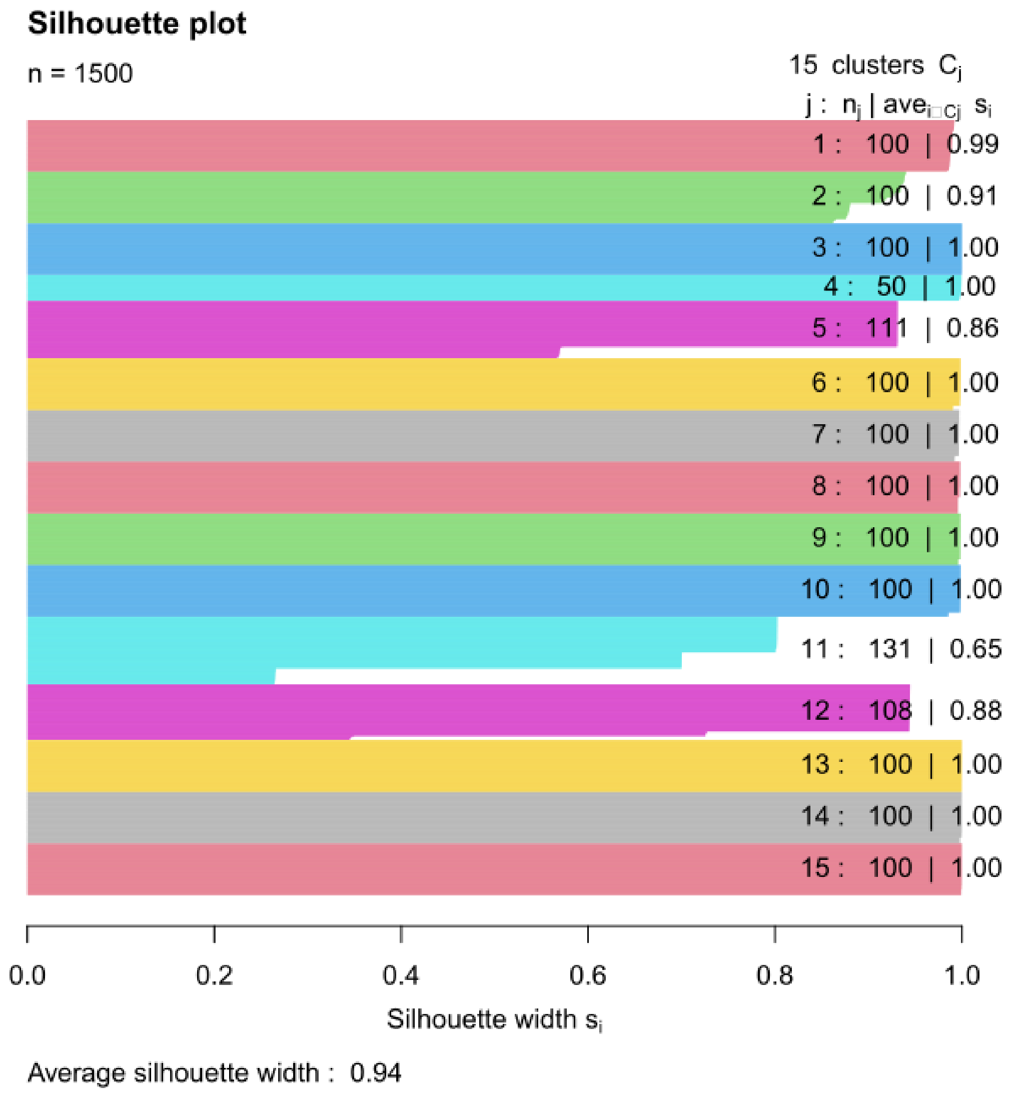

Fig G. *Reproducibility of components for protein representation. The consecutive bars of same color indicate the silhouette statistic of components within the same cluster. For each cluster, the numbers on the right indicate the cluster id, the cluster size and the average silhouette. The average silhouette among all the clusters (reproducibility score) is shown on the bottom.*

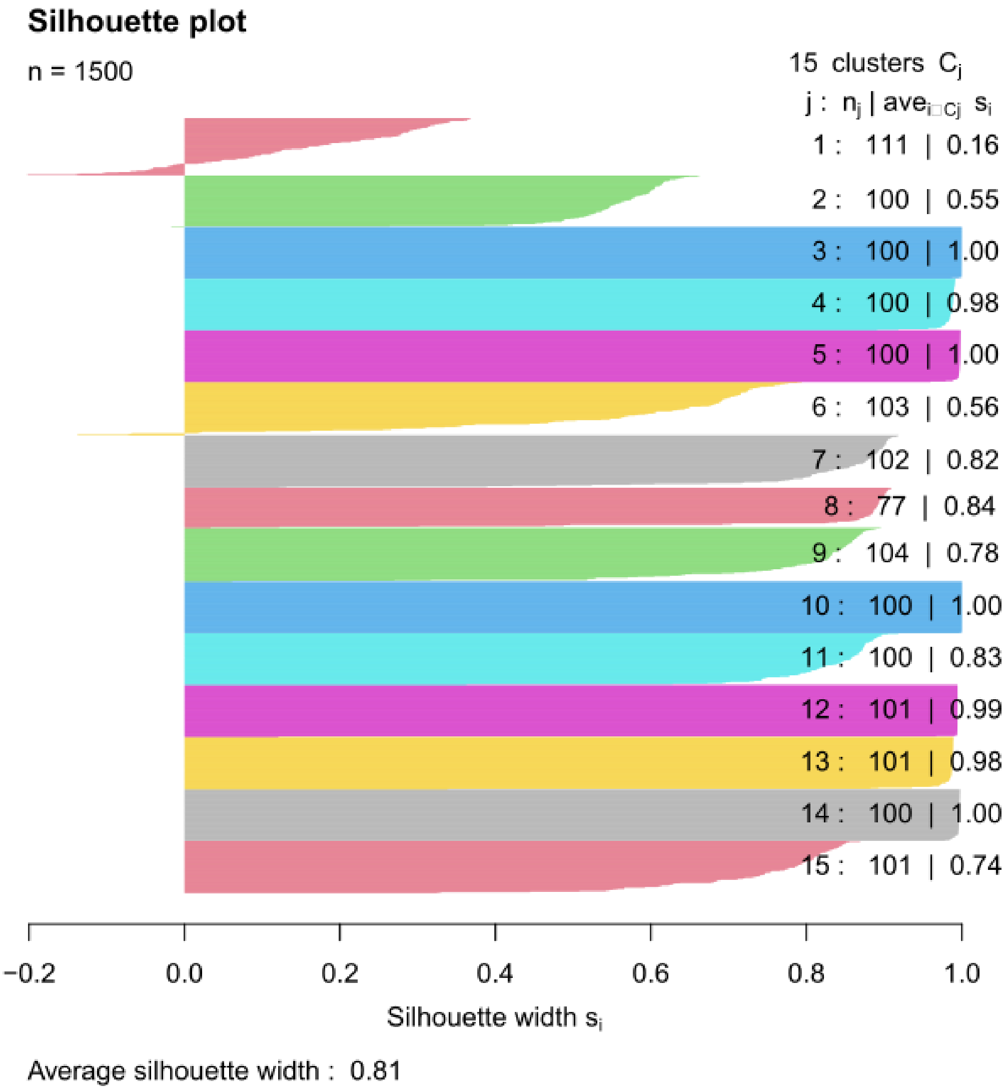

Fig H*. Reproducibility of components for gene representation. The consecutive bars of same color indicate the silhouette statistic of components within the same cluster. For each cluster, the numbers on the right indicate the cluster id, the cluster size and the average silhouette. The average silhouette among all the clusters (reproducibility score) is shown on the bottom.*

Note G. Relation between drug target signature and functional similarity for cellular components and molecular function GO categories

Fig. I-A and I-B show that drug target signature similarity is significantly correlated with functional similarity for cellular components (Spearman's correlation coefficient = 0.332, p-value = 0.0007), and molecular function (Spearman's correlation coefficient = 0.546, p-value = 4.093×10^{-9}) Gene Ontology (GO) categories, respectively. To measure functional similarity, we use semantic similarities between GO terms.

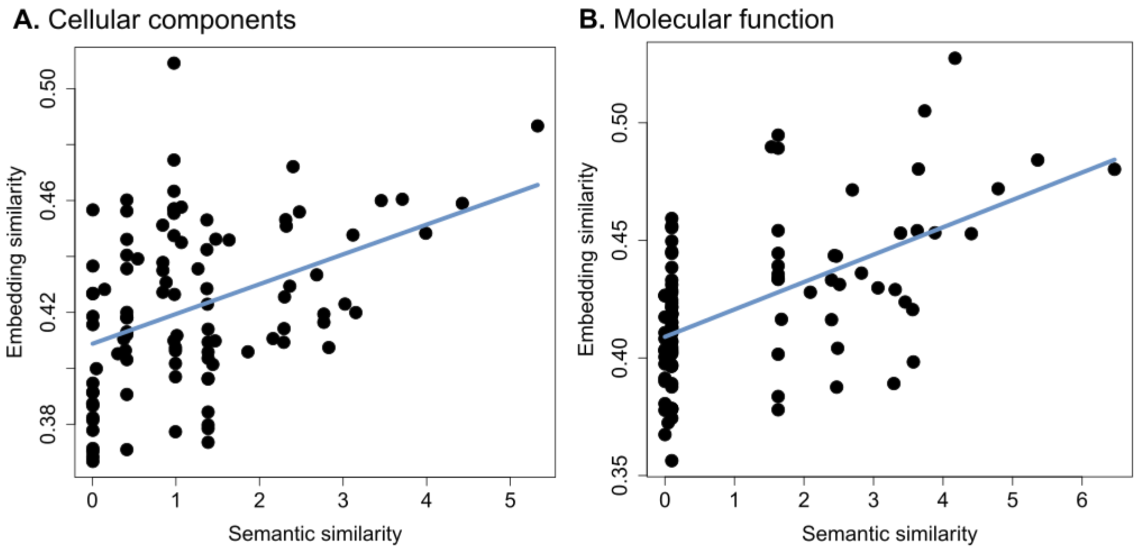

***Fig I**. Relation between drug target signature and semantic functional similarity. In each plot, protein pairs were ordered according to the value of their semantic similarity and then divided into 100 groups of equal size. Each group is represented by a dot, whose y-coordinate is the average signature between proteins of that group and the x-coordinate is the average semantic according to the Gene Ontology terms. Panels A and B show the results for cellular components and molecular function GO categories, respectively. For both GO categories, we found a positive and significant correlation between signature similarity and functional similarity.*

Note H. Top predictions for dengue and ebola viruses with in-vitro or in-vivo antiviral activity

Table B. *Drugs ranked among the top 100 by our method for predicted activity against Dengue virus (DENV) and Ebola virus (EBOV) infections, which have been shown to exhibit in vitro or in vivo efficacy.*

**Virus**	**Drug**	**Position in our ranking**	**Type of evidence**	**PubMed ID / Clinical Trials ID**
DENV	DB02709 - Resveratrol	9 (DENV-1),<br>9 (DENV-3),<br>9 (DENV-4),<br>11 (DENV-2),	In-vitro	PMID: 28216632
DENV	DB01254 - Dasatinib	17 (DENV-1),<br>17 (DENV-4),<br>18 (DENV-3),<br>21 (DENV-2)	In-vitro	PMID: 23616652,<br>PMID: 17360676
DENV	DB00563 - Methotrexate	38 (DENV-2),<br>45 (DENV-1),<br>45, (DENV-3),<br>48 (DENV-2)	In-vitro	PMID: 23824813
DENV	DB00458 - Imipramine	59 (DENV-2),<br>61 (DENV-1),<br>65 (DENV-3),<br>71 (DENV-4)	In-vitro	PMID: 28600536
DENV	DB00091 - Cyclosporine	63 (DENV-2),<br>81 (DENV-3),<br>91 (DENV-1),<br>103 (DENV-4)	In-vitro	PMID: 19451286
DENV	DB00481 - Raloxifene	66 (DENV-4),<br>70 (DENV-1),<br>74 (DENV-3),<br>100 (DENV-2)	In-vitro	PMID: 32482672
DENV	DB06616 - Bosutinib	64 (DENV-4),<br>72 (DENV-1),<br>72 (DENV-3),<br>98 (DENV-2)	In-vitro	PMID: 33683474
DENV	DB00908 - Quinidine	81 (DENV-2),<br>88 (DENV-1),	In-vitro	PMID: 30055216

		94 (DENV-3),<br>98 (DENV-4)		
EBOV	DB00661 - Verapamil	7	In-vitro	PMID:25722412
EBOV	DB00481 - Raloxifene	15	In-vitro	PMID:23785035,<br>PMID: 31964466
EBOV	DB00675 - Tamoxifen	23	In-vitro	PMID:23785035
EBOV	DB01076 - Atorvastatin	31	In-vitro	PMID: 29717011
EBOV	DB08860 - Pitavastatin	32	In-vitro	PMID: 29717011
EBOV	DB00227 - Lovastatin	44	In-vitro	PMID: 29717011
EBOV	DB01098 - Rosuvastatin	47	In-vitro	PMID: 29717011
EBOV	DB00255 - Diethylstilbestrol	48	In-vitro	PMID: 23785035
EBOV	DB00641 - Simvastatin	65	In-vitro	PMID: 29717011
EBOV	DB00619 - Imatinib	77	In-vitro	PMID: 22378924
EBOV	DB00338 - Omeprazole	79	In-vitro	PMID: 26069727
EBOV	DB01268 - Sunitinib	82	In vitro, Animal model, clinical trials	PMID:28240606;<br>NCT02380625
EBOV	DB00530 - Erlotinib	91	In vitro, Animal model, clinical trials	PMID: 28240606,<br>NCT02380625

EBOV	DB01050 - Ibuprofen	94	In-vitro	PMID: 28930583

Note I. AUC for viruses LASV, HAdV-2, MARV, HAdV-19, FLUBV, EMCV, HCoV-NL63, and EV-D68

Table C. *Cases where our method achieved AUC greater than 0.5 while the standard network medicine performed below this threshold.*

Virus	Standard network medicine approach	Our method
LASV	0.46	0.59
HAdV-2	0.19	0.59
MARV	0.46	0.63
HAdV-19	0.11	0.69
FLUBV	0.37	0.55
EMCV	0.39	0.88
HCoV-NL63	0.43	0.75
EV-D68	0.18	0.53

Note J. Gene expression data

We downloaded gene expression data from Gene Expression Omnibus - GEO (https://www.ncbi.nlm.nih.gov/geo/). Each downloaded dataset contains tissues/cell lines infected with a given virus and a group of controls/non-infected cells.

We downloaded normalized data for a few datasets, when it was available. For the non-normalized RNAseq data, we used edgeR R package for normalizing raw counts. For Illumina microarrays, we used the limma package for normalization. We pre-processed and normalized gene expression data from Affymetrix microarrays with function justRMA from affy package. All three R packages are available from Bioconductor R repository (https://www.bioconductor.org/).

In Table D, we show, for each virus, the GEO accession number, the sample type (tissue or cell line), the platform (RNAseq or microarray data), the sample size (number of infected samples and controls), whether the downloaded data is normalized or not, the PubMed ID (PMID) of the paper describing the data, and additional observations.

Table D. *Description of datasets downloaded from GEO containing gene expression data.*

Virus	GEO	Sample type	Platform	#Infected	#Controls	Norm. data available	PMID	Observations
CMV	GSE152114	Nasal turbinate tissues (in vitro culture)	RNAseq	5	5	Yes	32727881	
DENV-2	GSE128303	Primary human monocyte-derived macrophages (in vitro culture)	RNAseq	4	4	Yes	31306674	
EBOV	GSE100839	ARPE-19 cell (in vitro culture)	RNAseq	3	3	No	28721309	
EBV	GSE19761	Akata Burkitt's lymphoma clones (in vitro-culture)	Microarray (affymetrix)	3	3	No	23891576	
EV-A71	GSE71673	SK-N-SH cells (in vitro-culture)	Microarray (affymetrix)	3	3	No	28724943, 29263272	We selected 48hpi samples
H1N1	GSE89008	Human tracheobronchial epithelial (HTBE) cells (in vitro-culture)	RNAseq	2	2	Yes	30146161	We selected 3hpi samples
H3N2	GSE89008	Human tracheobronchial epithelial (HTBE) cells (in vitro-culture)	RNAseq	2	2	Yes	30146161	We selected 3hpi samples
H5N1	GSE89008	Human tracheobronchial epithelial (HTBE) cells (in vitro-culture)	RNAseq	2	2	Yes	30146161	We selected 3hpi samples
H7N9	GSE97949	A549 cells (in vitro culture)	RNAseq	2	2	Yes	29363430	We selected 3hpi samples
H9N2	GSE31471	A549 cells (in vitro culture)	Microarray (affymetrix)	3	3	No	22470468, 29104227, 32075271	We selected 2hpi samples

HBV	GSE135860	Huh7.5.1 cells (in vitro culture)	RNAseq	3	3	Yes	34725333	
HCV	GSE143300	Huh7 hepatoma cells (in vitro cell culture)	RNAseq	3	3	Yes	32764824	
HEV-3	GSE88731	HepG2/C3A cells (in vitro culture)	RNAseq	4	4	Yes		
HIV-1	GSE167098	HEK 293T cells (in vitro culture)	RNAseq	3	3	Yes		We selected 2hpi samples
HIV-2	GSE167098	HEK 293T cells (in vitro culture)	RNAseq	3	3	Yes		2 hpi
HMPV	GSE136139	A549 cell lines (in vitro culture)	RNAseq	2	2	No	32015498	We selected 2hpi samples
HSV-1	GSE103763	Human fibroblast KMB17 cells (in vitro culture)	RNAseq	3	3	Yes	29972822	
HTLV-1	GSE29312	whole blood gene expression profiles	Microarray (Illumina)	10	9	Yes	22291590	The infected group corresponds to HTLV-1-associated meylopathy
JCV	GSE45639	SVG 10B1 clones	Microarray (affymetrix)	3	3	No	24052414	We selected 10B1 clon, wheere the virus replicates the most
JEV	GSE39740	whole blood gene expression profiles	Microarray (affymetrix)	3	3	No	24069471	
LASV	GSE41300	PBMC cells (in vitro culture)	Microarray (affymetrix)	9	9	No	24069471	We selected 4hpi samples
LCMV	GSE12254	Liver from Macaques	Microarray (affymatrix)	11	3	No	19216742	
MeV	GSE980	Human CD14+ monocytes (in vitro culture)	Microarray (affymatrix)	3	4	No	16492729	We selected 6hpi samples
ORFV	GSE93226	Human foreskin fibroblast (HFF-1) cells (in vitr culture)	RNAseq	3	3	No	28938587	We selected 3hpi samples
RABV	GSE119636	Human neuroblastoma cells (in vitro culture)	Microarray (Agilent)	3	3	No	30391720	
RSV	GSE166161	Nasal curettage cells	RNAseq	18	8	No	33795879	
RVFV	GSE102481	HSAEC cells (in vitro culture)	RNAseq	3	3	No	29408900	We selected 9hpi samples
SARS-CoV	GSE30589	ΔE-infected cells (in vitro culture)	Microarray (affymatrix)	3	3	No	22028656	
SARS-CoV-2	GSE152075	Profiles of nasopharyngeal swabs from 430 individuals with SARS-CoV-2 and 54 negative controls. RNAseq	RNAseq	430	54	No	32898168	
SINV	GSE125182	HEK293 cells (in vitro culture)	RNAseq	3	3	No	30799147	We selected 4hpi samples
VACV	GSE165732	HCT-116 cells (in vitro culture)	RNAseq	3	3	No	33912921	

VZV	GSE175797	human brain vascular adventitial fibroblasts - HBVAFs (in vitro culture)	Microarray (affymatrix)	3	3	No	34759019	
WNV	GSE46681	Human PBMCs	Microarray (Illumina)	39	39	No	25355795	
ZIKV	GSE131605	Human iris pigment epithelial cell (in vitro culture)	RNAseq	20	20	No	33968035	

Note K. Results for different values of $\lambda_1, \lambda_2, \lambda_3, \lambda_4$

Our model has four hyper parameters ($\lambda_1, \lambda_2, \lambda_3, \lambda_4$) that control the contribution of the regularization terms.

To verify how sensitive our model is to these hyper parameters, we ran our method with different values of $\lambda_1, \lambda_2, \lambda_3, \lambda_4$. We considered two scenarios. In the first one, all four parameters have a same value a, that is $\lambda_1 = \lambda_2 = \lambda_3 = \lambda_4 = a$. We varied a between 0 and 1. In the second scenario the parameters can assume two different values $b_1$and b_2 between 0 and 1, with $\lambda_1 = \lambda_4 = b_1$, and $\lambda_2 = \lambda_3 = b_2$.

The aim of the second scenario is evaluating the impact of having different weights for the regularization terms. Because the number of possible combinations is high, we focused on the particular case where λ_1 (regularization term for the drug representation) is bigger than λ_2(regularization term for the virus representation). In that case, we try to compensate the fact that the number of drugs is much higher than the number of viruses by adding a stronger regularization for the drug representation. We also considered λ_4 (regularization term for the gene representation) bigger than λ_3 (regularization term for the proteins) for compensating the fact that in our dataset the number of genes is higher than the number of proteins.

We show the area under the ROC curve (AUC) in both scenarios in Fig J and Fig K respectively. Each boxplot in Fig J corresponds to a different value of a and each boxplot in Fig K corresponds to different values of $b_1$and b_2. The median AUC varies between 0.697 and 0.728 (standard deviation of the medians = 0.016) in the first scenario, and between 0.678 and 0.718 (standard deviation of the medians = 0.017) in the second scenario. In comparison, the standard deviation of the median AUC when running our model with default parameters 200 times was 0.015. Thus, this analysis suggests that our model is not particularly sensitive to $\lambda_1, \lambda_2, \lambda_3, \lambda_4$.

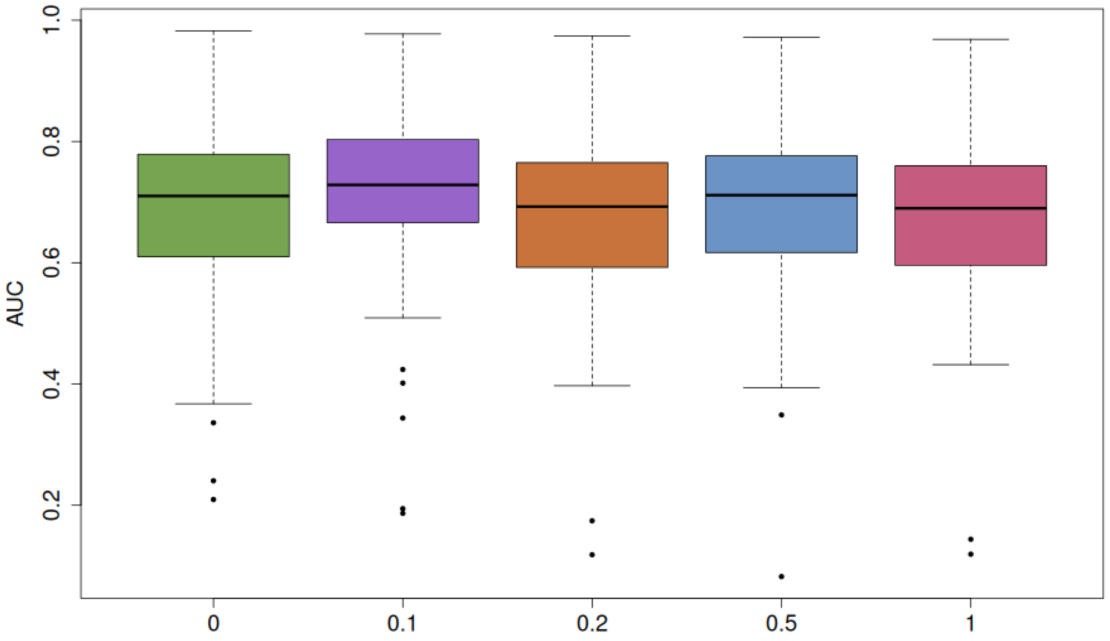

Fig J. *Area under the ROC curve for different values of* $\boldsymbol{a}$*, where* $\boldsymbol{\lambda_1} = \boldsymbol{\lambda_2} = \boldsymbol{\lambda_3} = \boldsymbol{\lambda_4} = \boldsymbol{a}$*. For each of the 55 viruses considered for evaluation, we obtained a ranking of drugs according to their predicted efficacy score. We ran our model for a = 0, 0.1, 0.2, 0.5, 1. Each boxplot shows the area under the ROC curve across viruses for a given values of* $\boldsymbol{a}$*.*

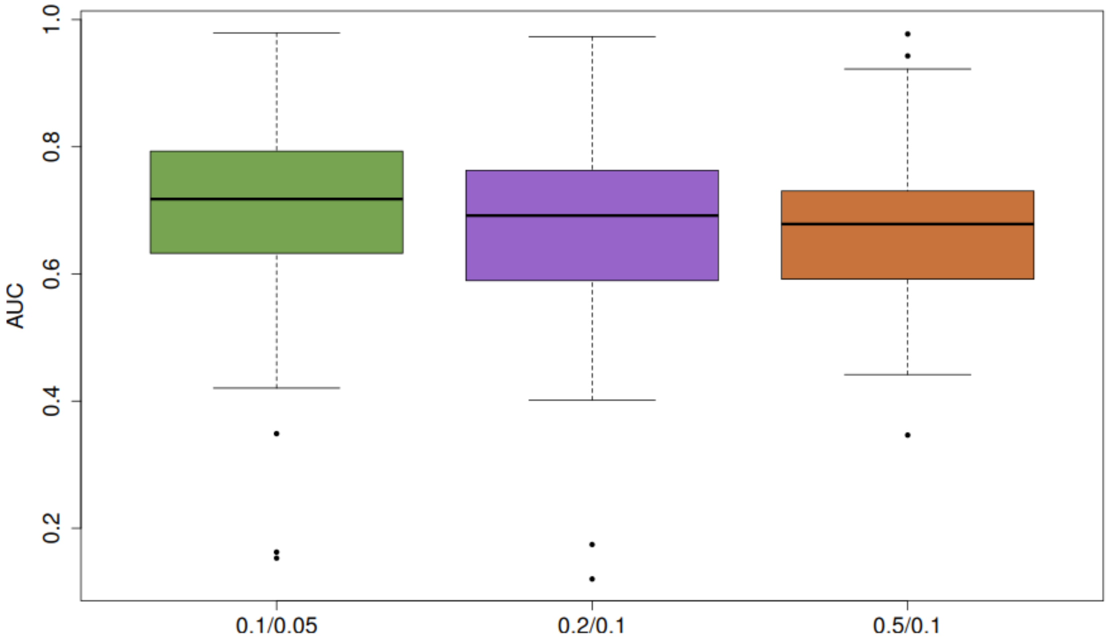

Fig K. *Area under the ROC curve for different values of* $\boldsymbol{b_1}$*and* $\boldsymbol{b_2}$*, where* $\boldsymbol{\lambda_1} = \boldsymbol{\lambda_4} = \boldsymbol{b_1}$ *and* $\boldsymbol{\lambda_2} = \boldsymbol{\lambda_3} = \boldsymbol{b_2}$*. For each of the 55 viruses considered for evaluation, we obtained a ranking of drugs according to their predicted efficacy score. We ran our model for* $(\boldsymbol{b_1}, \boldsymbol{b_2}) = (\mathbf{0.1}, \mathbf{0.05}), (\mathbf{0.2}, \mathbf{0.1}), (\mathbf{0.5}, \mathbf{0.1})$*. Each boxplot shows the area under the ROC curve across viruses for given values of* $\boldsymbol{b_1}/\ \boldsymbol{b_2}$*.*

References

5. Santos, S. de S. et al. Machine learning and network medicine approaches for drug repositioning for COVID-19. Patterns (N Y) 3, 100396 (2022).

15. Gysi, D. M. et al. Network medicine framework for identifying drug-repurposing opportunities for COVID-19. Proc. Natl. Acad. Sci. 118, (2021).

16. Fiscon, G., Conte, F., Farina, L. & Paci, P. SAveRUNNER: A network-based algorithm for drug repurposing and its application to COVID-19. PLoS Comput. Biol. 17, e1008686 (2021).

17. Guney, E., Menche, J., Vidal, M. & Barábasi, A.-L. Network-based in silico drug efficacy screening. Nat. Commun. 7, 10331 (2016).

24. Li, Z. et al. A computational framework of host-based drug repositioning for broad-spectrum antivirals against RNA viruses. iScience 24, 102148 (2021).

25. Li, Z., Yao, Y., Cheng, X., Li, W. & Fei, T. An in silico drug repositioning workflow for host-based antivirals. STAR Protocols 2, 100653 (2021).

56. Smola, A. J. & Kondor, R. Kernels and Regularization on Graphs. in Learning Theory and Kernel Machines (eds. Schölkopf, B. & Warmuth, M. K.) 144–158 (Springer, Berlin, Heidelberg, 2003). doi:10.1007/978-3-540-45167-9_12.

57. Picart-Armada, S., Thompson, W. K., Buil, A. & Perera-Lluna, A. diffuStats: an R package to compute diffusion-based scores on biological networks. Bioinformatics 34, 533–534 (2018).
